# Pseudohypoxic stabilization of HIF1α via cyclophilin D suppression promotes melanoma metastasis

**DOI:** 10.1038/s41392-025-02314-8

**Published:** 2025-07-24

**Authors:** Hye-Kyung Park, Sung Hu, So Yeon Kim, Sora Yoon, Nam Gu Yoon, Ji Hye Lee, Wonyoung Choi, Sun-Young Kong, Jong Heon Kim, Dougu Nam, Byoung Heon Kang

**Affiliations:** 1 Department of Biological Sciences, Ulsan National Institutes of Science and Technology (UNIST), Ulsan, South Korea; 2 Department of Genetics, University of Pennsylvania, Philadelphia, PA USA; 3 Department of Cancer Biomedical Science, National Cancer Center Graduate School of Cancer Science and Policy, Goyang, South Korea; 4 Center for Clinical Trials, National Cancer Center, Goyang, South Korea; 5 Department of Laboratory Medicine, National Cancer Center, Goyang, South Korea; 6 Cancer Molecular Biology Branch, Research Institute, National Cancer Center, Goyang, South Korea

**Keywords:** Metastasis, Molecular medicine

## Abstract

Stabilization of hypoxia-inducible factor 1 alpha (HIF1α), which plays a pivotal role in regulating cellular responses to insufficient oxygen, is implicated in cancer progression, particularly epithelial-mesenchymal transition and metastatic dissemination. Despite its crucial role in tumorigenesis, the precise mechanisms governing HIF1α stabilization under varying tumor microenvironmental conditions are not fully understood. In this study, we show that stabilization of HIF1α in metastasizing melanoma under mild hypoxia is regulated primarily by mitochondrial reactive oxygen species (ROS) rather than by reduced oxygen levels. Activated HIF1α suppresses the expression of cyclophilin D (CypD), a regulator of the mitochondrial permeability transition pore (mPTP), as a reciprocal regulatory mechanism to sustain HIF1 signaling via upregulation of microRNAs miR-23a and miR-27a. Reduced expression of CypD leads to mPTP closure, resulting in elevated mitochondrial calcium accumulation and enhanced oxidative phosphorylation, which in turn increases mitochondrial ROS levels. The ROS then inhibits a prolyl hydroxylase, establishing a pseudohypoxic state that stabilizes HIF1α even in the presence of oxygen. This HIF1-reinforced and mitochondria-driven pseudohypoxic induction is essential for maintaining HIF1 signaling under conditions of mild hypoxia or transient increases in oxygen levels during melanoma metastasis. Overexpression of CypD reversed the pseudohypoxic state and potently inhibited melanoma metastasis. Thus, mitochondria-driven pseudohypoxic induction is critical for sustaining HIF1 signaling in metastasizing cancer cells and can be exploited to develop anti-metastatic therapies.

## Introduction

Cancer begins as a locoregional disease; however, the primary tumor eventually metastasizes to lymph nodes and distal organs, which is the main cause of cancer-associated death.^1^ Metastasis of cancer cells from their epithelial origins is initiated by switching on the developmentally programmed process of epithelial-mesenchymal transition (EMT).^2–4^ The complex and coordinated multistep processes regulating EMT, and subsequent generation of metastatic cancer cells with mesenchymal features, are influenced significantly by the tumor microenvironment (TME) surrounding the primary tumor; one of the main drivers of EMT is hypoxia.^5,6^

Rapidly growing solid tumors often experience oxygen shortages, resulting in a hypoxic state within the TME. Hypoxia is a prominent characteristic of the TME, and is closely associated with acquisition of invasive, migratory, and drug-resistant properties by cancer cells within the primary tumor, resulting in metastasis.^7^ Hypoxia often activates hypoxia-inducible factors (HIFs) such as HIF1α, as part of the cellular adaptive response; HIFs have the ability to increase the metastatic properties of cancer cells.^5,7^

Prolyl hydroxylases (PHDs) regulate HIF1α levels according to oxygen availability. PHDs hydroxylate HIF1α in an oxygen-dependent manner, leading to polyubiquitination and proteasomal degradation under normoxic TME conditions,^8,9^ whereas a hypoxic TME inhibits PHD due to insufficient oxygen, thereby stabilizing the transcription factor.^10^ Additionally, PHD can be inactivated independently of the oxygen concentration, leading to aerobic stabilization of HIF1α, referred to as pseudohypoxic conditions; such conditions can arise from changes to the production of various metabolites, including reactive oxygen species (ROS).^11,12^ Hypoxic and pseudohypoxic activation of HIF1α is often crucial for facilitating EMT and subsequent metastasis of cancer cells; however, a comprehensive understanding of the oxygen-dependent or independent activation of HIF1α, particularly in the context of metastasis, remains elusive.

The mitochondrial permeability transition pore (mPTP) plays an important role in non-selective transport of small molecules, including Ca^2+^, across mitochondrial membranes. Cyclophilin D (CypD, *PPIF*), a mitochondrial matrix peptidyl-prolyl *cis-trans* isomerase, primarily regulates opening of the mPTP,^13,14^ which is formed by adenine nucleotide translocase (ANT) and F_1_F_O_-ATP synthase in the mitochondrial inner membrane.^15^ Opening of the mPTP redistributes ions and metabolites across the mitochondrial inner membrane, subsequently altering a variety of metabolic and signaling pathways;^16,17^ therefore, activation of CypD and the opening of mPTP are thought to have a significant impact on tumorigenesis.^18,19^ However, there is currently no consensus regarding the effects of the mPTP on tumorigenesis, as conflicting findings suggest both supportive and suppressive effects.

Here, we show that reduced CypD activity results in closure of the mPTP, and that this process is crucial for stabilizing HIF1α through an oxygen-independent mechanism mediated by ROS under conditions of mild hypoxia (in which oxygen levels remain sufficient for PHD enzyme activity). This pseudohypoxic state, which sustains HIF1α signaling, is essential for promoting EMT and metastasis of cancer cells. Consequently, an increase in CypD levels disrupts the pseudohypoxic state and potently inhibits cancer metastasis, thereby offering a promising strategy for anti-metastatic therapy.

## Results

### Reduced expression of CypD is associated with a poor prognosis for melanoma patients and with increased metastatic potential

**Fig. 1:**
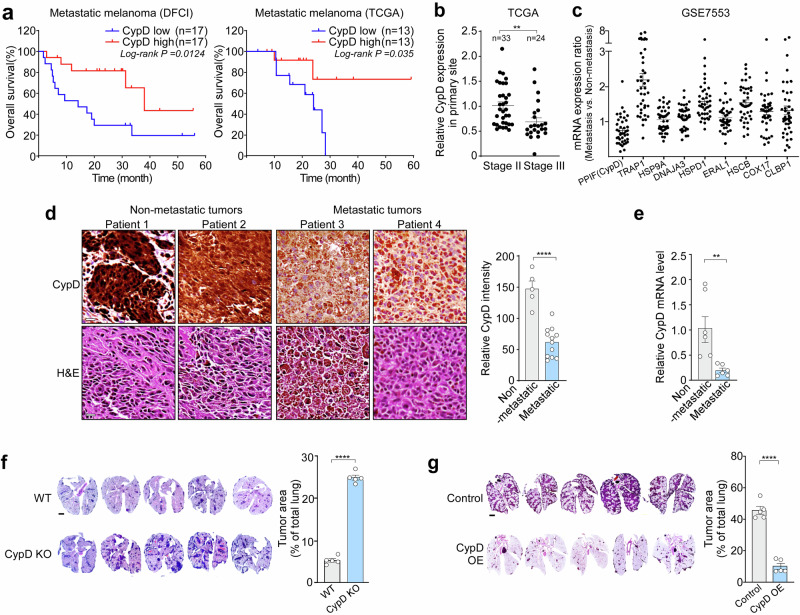
CypD expression and melanoma metastasis. **a** CypD expression and overall survival of melanoma patients with metastasis. DFCI^86^ and TCGA^87^ data were downloaded from the cBioPortal database (http://www.cbioportal.org).^20,21^ Overall survival rates in the high CypD expression group (top 25% of patients) and low CypD expression group (bottom 25% of patients) were analyzed and plotted as Kaplan-Meier curves. **b** Expression of CypD in non-metastatic (Stage II) and metastatic melanoma patients (Stage III). TCGA^88^ data were obtained from the cBioPortal database and compared with respect to CypD expression. **c** Expression of mitochondrial chaperones in metastatic tumors. Datasets from patients with metastatic (n = 41) and non-metastatic (n = 40) skin cancer were obtained from the Gene Expression Omnibus database^84^ (GSE7553), and mRNA levels of mitochondrial chaperones were compared. The results are presented as dot plots. **d** Immunohistochemical (IHC) and hematoxylin & eosin(H&E) staining of primary tumor tissues from melanoma patients. H&E and IHC staining using a CypD antibody were performed on non-metastatic (n = 5) and metastatic primary tumors (n = 12) (left). CypD expression was quantified using ImageJ (right). Scale bar, 20 μm. **e** CypD mRNA expression in melanoma patients. Total RNA was extracted from non-metastatic (n = 3) and metastatic primary tumors (n = 3), followed by RT-qPCR analysis. Relative CypD mRNA levels were compared between the two group. **f** Lung-infiltrating metastasis of CypD knockout (KO) cells. Wild-type (WT) or CypD KO B16F10 cells (1 × 10^5^ cells) were injected into the tail vein of C57BL/6 mice, and the lungs were harvested and analyzed by H&E staining (left). Tumor area was quantified using ImageJ software to examine the metastatic burden (right, n = 5 per group). The scale bar represents 1 mm. **g** Lung-infiltrating metastases of CypD-overexpressing (OE) cells. B16F10 cells (5 × 10^5^ cells) stably transfected with pcDNA (Control) or pcDNA-CypD (CypD OE) were injected into the tail vein of C57BL/6 mice (n = 5), and analyzed as in (**d**). Scale bar, 1 mm. Data are presented as the mean ± SEM. **, *p* < 0.01; ****, *p* < 0.0001

To comprehend the function of mPTP in tumorigenesis, we investigated whether expression of CypD, the regulator of mPTP opening,^16^ affects survival of melanoma patients. Analysis of RNA sequencing (RNA-seq) data from melanoma patients with metastases obtained from the cBioPortal database (http://www.cbioportal.org)^20,21^ revealed that patients with low expression of CypD in the primary tumor have a poorer prognosis than those with high expression (Fig. 1a). Furthermore, patients with stage III melanoma that metastasized to the lymph nodes showed lower expression of CypD than those with stage II melanoma without metastasis (Fig. 1b). These results suggest that lower expression of CypD is associated with poorer survival rate, potentially due to increased metastasis. Notably, in the GEO dataset (GSE7553), which includes both metastatic and non-metastatic cancer samples, CypD was the only mitochondrial chaperone whose expression was reduced (by 30%). In contrast, the expression of other chaperones in metastatic tumors was either higher or comparable to that in non-metastatic tumors (Fig. 1c). To further validate the clinical relevance of CypD regulation in melanoma metastasis, we obtained melanoma patient samples with and without metastases and performed immunohistochemistry (IHC) and RT-PCR analyses. Our results confirmed that both CypD protein and mRNA expression levels were significantly lower in metastatic melanoma samples with poor prognosis compared to non-metastatic cases (Fig. 1d, e).

To elucidate the contribution of reduced CypD expression to metastasis, the *Ppif* gene, encoding CypD, was deleted from mouse melanoma B16F10 cells. Subsequently, CypD knockout (KO) cells were injected into C57BL/6 mice through the tail vein to assess their potential for lung metastasis. The CypD KO cells led to a 3-fold increase in metastatic foci on the lung surfaces (Supplementary Fig. 1a), and exhibited a 5-fold increase in lung-infiltrating metastases than control cells (Fig. 1f); however, CypD KO did not affect cell proliferation in vitro or tumor growth in vivo (Supplementary Fig. 1b, c). Conversely, stable overexpression (OE) of CypD resulted in a 5-fold reduction in lung metastasis when compared with control B16F10 cells transfected with pcDNA (Control) (Fig. 1g; Supplementary Fig. 1d), with no significant effect on cell proliferation in vitro or tumor growth in vivo (Supplementary Fig. 1e, f). Interestingly, tumors derived from CypD KO cells exhibited increased expression of pro-metastatic genes such as *Il6*, *Tnf*, *Tgfb1*, *Vegfa*, *Angptl4*, *Loxl2*, *Mmp2*, and *Mmp9*, which are involved in inflammation, angiogenesis, and extracellular matrix remodeling. This effect was fully reversed in CypD OE cells (Supplementary Fig. 1g). Furthermore, RNA-seq analysis revealed that CypD OE significantly downregulated both glycolytic and oxidative phosphorylation pathways (Supplementary Fig. 1h), indicating impaired metabolic adaptation.

Collectively, these findings suggest that CypD expression may influence the survival of melanoma patients not through regulation of primary tumor growth, but rather through modulation of the metastatic potential of cancer cells.

### Ablating CypD activates the HIF1 and EMT pathways

**Fig. 2:**
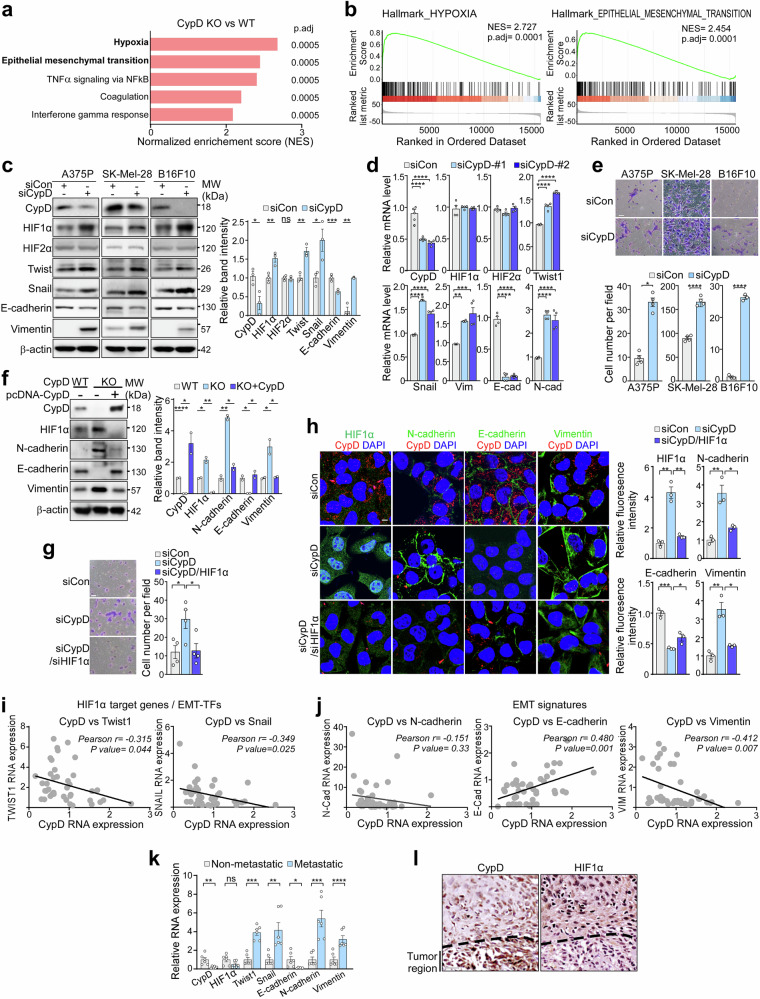
Upregulation of hypoxia and EMT pathways after suppression of CypD. **a** Gene set enrichment analysis (GSEA).^83^ WT and CypD KO B16F10 cells cultured under normoxia were analyzed by RNA-seq. The hallmark pathways upregulated in CypD KO compared with WT B16F10 cells were presented using normalized enrichment scores (NES). The p-value adjusted using the Benjamini-Hochberg procedure (p.adj) was indicated. **b** Enrichment scores for the two highest-ranking pathways were plotted. **c** Expression of EMT markers and HIF1α after CypD knockdown (KD). A375P, SK-Mel-28, and B16F10 cells were treated for 24 h with control siRNA (siCon) or CypD siRNA (siCypD), and then analyzed by western blotting. **d** Analysis of mRNA after CypD KD. Total RNA was isolated from siRNA-treated A375P cells and then analyzed by reverse transcription-quantitative PCR (RT-qPCR, n = 4). **e** Cell invasion after CypD KD. The invasive capacity of A375P, SK-Mel-28, and B16F10 cells following treatment of siCon or siCypD was assessed for 24 h in a Transwell invasion assay. Scale bar, 50 µm. The number of invaded cells was quantified (n = 4). **f** Expression of EMT markers and HIF1α after CypD overexpression in CypD KO cells. WT or CypD KO B16F10 cells were transfected for 24 h with pcDNA (-) or pcDNA-CypD (+), and then analyzed by western blotting (left). Band intensity was quantified using ImageJ (right). **g** Cell invasion after knockdown of CypD and HIF1α. A375P cells were transfected with siRNA targeting CypD or HIF1α (n = 4), and then analyzed as in (**e**). Scale bar, 50 µm. **h** EMT markers after knockdown of CypD and HIF1α. A375P cells treated with control, CypD, or HIF1α siRNAs were subjected to immunofluorescence analysis (left). Fluorescence intensity was quantified using ImageJ (right, n = 4). Scale bar, 5 µm. **i** Correlation between expression of CypD and that of EMT transcription factors (Twist1 and Snail) in melanoma patients with metastatic disease. RNA-seq datasets obtained from the Gene Expression Omnibus database^84^ (GSE7553) were analyzed. **j** Correlation between expression of CypD and EMT markers (E-cadherin, N-cadherin, and vimentin) in melanoma patients with metastatic disease was analyzed as in (**i**). **k** mRNA expression in patient samples. Total RNA was extracted from non-metastatic (n = 3) and metastatic primary tumors (n = 3) and analyzed by RT-qPCR. CypD mRNA levels were compared between the two groups. **l** IHC staining of primary tumor tissues from melanoma patients. IHC staining were performed on metastatic primary tumor tissues using an anti-CypD and anti-HIF1α antibody. Scale bar, 20 μm. All data are presented as the mean ± SEM. *, *p* < 0.05; **, *p* < 0.01; ***, *p* < 0.001, ****, *p* < 0.0001; ns, not significant

To investigate the regulatory mechanisms underlying the effect of CypD on metastasis, we conducted RNA-seq experiments on WT and CypD KO B16F10 cells cultured under normal oxygen concentrations (21% O_2_). To our surprise, pathway analysis of RNA-seq data (GSEA) indicated that among the Hallmark pathway collection, “hypoxia” and “epithelial-mesenchymal transition (EMT)” were the two most upregulated pathways in CypD KO cells compared with WT cells (Fig. 2a, b; Supplementary Table 2). Consistent with this, we found that under normoxic conditions, knockdown (KD) of CypD by siRNAs increased HIF1α levels in human (A375P and SK-Mel-28) and mouse (B16F10) melanoma cells (Fig. 2c); however, inhibiting CypD did not affect HIF2α expression or HIF1α mRNA levels (Fig. 2c, d), suggesting specific regulation of HIF1α protein stability.

The hallmark features of EMT, including loss of E-cadherin expression and the gain of expression of N-cadherin, vimentin, Snail, and Twist, were confirmed in CypD KD cells under normoxic conditions (Fig. 2c, d; Supplementary Fig. 2a, b). Snail and Twist are key transcription factors that induce EMT by reducing cell adhesion and epithelium-maintaining signals through the regulation of several genes, including E-cadherin, N-cadherin, and vimentin expression.^22^ CypD KD led to increased invasiveness of both human and mouse melanoma cells (Fig. 2e). To establish a causal relationship between CypD inhibition, HIF1α stabilization, and EMT induction, we performed a rescue experiment by overexpressing CypD in CypD KO cells. The elevated levels of HIF1α and EMT markers in CypD KO cells were fully reversed upon CypD OE, confirming that CypD depletion directly induces HIF1α stabilization and EMT activation (Fig. 2f). Furthermore, inhibiting HIF1α by siRNA completely reversed both the gained EMT features and the increased cell invasiveness acquired after CypD inhibition (Fig. 2g, h; Supplementary Fig. 2c–e). Conversely, the anti-metastatic effect of CypD OE was fully reversed by overexpression of HIF1α Pro402A/Pro564A (HIF1α P2A), a degradation-resistant mutant^23,24^ (Supplementary Fig. 2f, g), further confirming that CypD inhibits metastasis by destabilizing HIF1α.

Analysis of data from metastatic melanoma patients (GSE7553) revealed that CypD expression correlated inversely with that of EMT transcription factors and EMT features (Fig. 2i, j). Similarly, analysis of primary melanoma tissues confirmed lower CypD and higher EMT marker levels in metastatic patient specimens compared to non-metastatic ones (Fig. 2k). Moreover, we observed that migrating cancer cells from primary tumor sites exhibited lower CypD expression and higher HIF1α expression in metastatic melanoma patients (Fig. 2l), further reinforcing the link between CypD suppression and metastatic potential. Collectively, these results strongly suggest that reduced expression of CypD is sufficient to increase metastasis by activating HIF1α-mediated EMT pathways, even under normoxic conditions.

### CypD deficiency increases mitochondrial calcium levels and ROS production by closing the mPTP

**Fig. 3:**
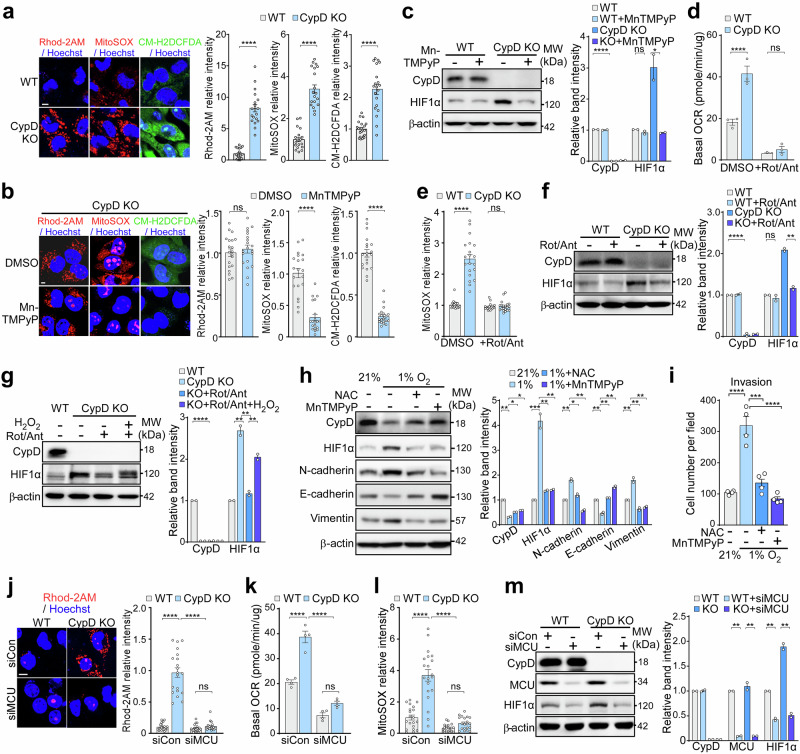
Increased ROS production due to mPTP closure and enhanced oxidative phosphorylation. **a** Mitochondrial calcium and ROS levels in CypD KO cells. WT or CypD KO B16F10 cells were stained with Rhod-2AM, MitoSOX, or CM-H2DCFDA to examine mitochondrial Ca^2+^, mitochondrial ROS, and cytosolic ROS level, respectively. The cells were analyzed by confocal microscopy (left; scale bar, 5 µm), and their fluorescence pixel values were compared (right; n = 20). **b** Mitochondrial calcium and ROS levels following MnTMPyP treatment. MnTMPyP-treated CypD KO B16F10 cells were analyzed as in (**a**). **c** HIF1α expression after MnTMPyP treatment in CypD KO cells. WT or CypD KO B16F10 cells were incubated with either DMSO or MnTMPyP for 6 h and then analyzed by western blotting (left). The band intensity was quantified using ImageJ (right). **d** Basal oxygen consumption rate (OCR) in WT and CypD KO B16F10 cells. The basal OCR of WT and CypD KO cells treated with rotenone/antimycin A (Rot/Ant, 2 μM for each) was measured using the Seahorse XF analyzer (n = 3). **e** Mitochondrial ROS levels in CypD KO cells after Rot/Ant treatment. WT or CypD KO B16F10 cells were treated with DMSO or Rot/Ant for 30 min, stained with MitoSOX, and analyzed by fluorescence microscopy. The fluorescence intensity was quantified using ImageJ (n = 30). **f** HIF1α expression in CypD KO cells after Rot/Ant treatment. WT or CypD KO B16F10 cells were treated with DMSO or Rot/Ant and then analyzed by western blotting (left). The band intensity was quantified (right). **g** HIF1α expression in CypD KO cells after H_2_O_2_ treatment. WT or CypD KO B16F10 cells were treated with DMSO, Rot/Ant, or 1 mM H_2_O_2_ and analyzed by western blotting (left). The band intensity was quantified (right). **h** Expression of EMT markers and HIF1α after ROS scavenger treatment under hypoxia. B16F10 cells were treated with DMSO, 1 mM NAC, or 20 μM MnTMPyP under hypoxia, and analyzed by western blotting (left). The band intensity was quantified (right). **i** Cell invasion after ROS scavenger treatment under hypoxia. B16F10 cells were treated with DMSO, 1 mM NAC, or 20uM MnTMPyP under hypoxia and analyzed for cell invasion as in Fig. 2e. **j** Mitochondrial calcium level in CypD KO cells after knockdown of mitochondrial calcium uniporter (MCU). WT or CypD KO cells were transfected with control siRNA (siCon) or siRNA targeting MCU (siMCU) for 24 h. Cells were then stained with Rhod-2AM and analyzed by confocal microscopy (left, scale bar: 10 µm). The fluorescence intensity was quantified (n = 20). **k** Basal oxygen consumption rate (OCR) in WT and CypD KO cells after MCU KD. The basal OCR of WT and CypD KO cells transfected with siCon or siMCU was measured using the Seahorse XF analyzer (n = 4). **l** Mitochondrial ROS levels in CypD KO cells following MCU KD. WT or CypD KO cells transfected with siCon or siMCU we analyzed as in (**e**) (n = 30). **m** HIF1α expression in CypD KO cells after MCU KD. WT or CypD KO cells were transfected with siCon or siMCU for 24 h and then analyzed by western blotting (left). The band intensity was quantified (right). Data are presented as the mean ± SEM. *, *p* < 0.05; **, *p* < 0.01; ***, *p* < 0.001, ****, *p* < 0.0001; ns, not significant

To elucidate the mechanism underlying pseudohypoxia, i.e., HIF1α activation under sufficient oxygen levels (normoxic conditions),^25^ upon reduction in CypD levels, we explored the relevance of mPTP opening, the key mitochondrial function of CypD that affects various metabolic and signaling pathways.^26^ CypD expression positively correlated with the TMRM flickering frequency, which reflects the opening frequency of mPTP (Supplementary Fig. 3a), indicating the presence of CypD-dependent, reversible, transient mPTP opening in melanoma cells as previously reported.^27^ In CypD KO melanoma cells, TMRM flickering was reduced, accompanied by increased accumulation of mitochondrial calcium, indicative of mPTP closure,^28^ along with increased generation of both mitochondrial and cellular ROS (Fig. 3a). Similarly, following thapsigargin treatment, mitochondrial calcium levels were reduced most rapidly in CypD OE, followed by WT, and were slowest in CypD KO cells, indicating that mPTP closure in the absence of CypD blocked calcium efflux from mitochondria (Supplementary Fig. 3b). Electron microscopy analysis showed that mitochondrial morphology remained unremarkable between WT, CypD KO, and CypD OE cells (Supplementary Fig. 4), further supporting that the observed phenotypic differences arise from functional rather than structural mitochondrial alterations.

MnTMPyP, a mitochondrial ROS scavenger, in CypD KO cells did not affect mitochondrial calcium levels, but it did reduce stabilization of HIF1α, as well as decreasing mitochondrial and cellular ROS levels (Fig. 3b, c), indicating that the increased ROS production caused by CypD deficiency was responsible for HIF1α stabilization. Production of ROS after CypD KO coincided with an increase in oxygen consumption (Fig. 3d); thus, inhibiting oxidative phosphorylation (OxPhos) by exposure to Complex I and III inhibitors rotenone and antimycin A reduced mitochondrial ROS levels to those in WT cells, and also reduced HIF1α expression (Fig. 3e, f). Conversely, the addition of H_2_O_2_ restored HIF1α levels in CypD KO cells with rotenone and antimycin A (Fig. 3g). Furthermore, both the mitochondrial ROS scavenger MnTMPyP and the cytosolic ROS scavenger N-acetylcysteine (NAC) effectively reversed the hypoxia-induced increase in HIF1α and EMT features, as well as the decrease in CypD expression, thereby significantly reducing the invasive capacity of cancer cells (Fig. 3h, i). The data suggest that elevated levels of mitochondrial Ca^2+^ in CypD KO cells activate OxPhos by increasing the activity of mitochondrial catabolic enzymes, which is consistent with the metabolic regulatory functions of mPTP,^29–32^, ultimately leading to elevated ROS production and subsequent HIF1α stabilization.

Given that mitochondrial calcium influx is primarily mediated by the mitochondrial calcium uniporter (MCU),^33,34^we inhibited MCU using either siRNA or ruthenium red (RR)^35^ to further elucidate the contribution of mitochondrial calcium to HIF1α stabilization. Inhibition of the MCU reduced accumulation of mitochondrial calcium, decreased oxygen consumption and ROS production, and induced degradation of HIF1α in CypD KO melanoma cells (Fig. 3j–m; Supplementary Fig. 5a–c). Collectively, stabilization of pseudohypoxic HIF1α after CypD inhibition is attributed to mPTP closure, which in turn affects mitochondrial OxPhos and production of ROS.

### HIF1α stabilization under conditions of mild hypoxia requires suppression of CypD

**Fig. 4:**
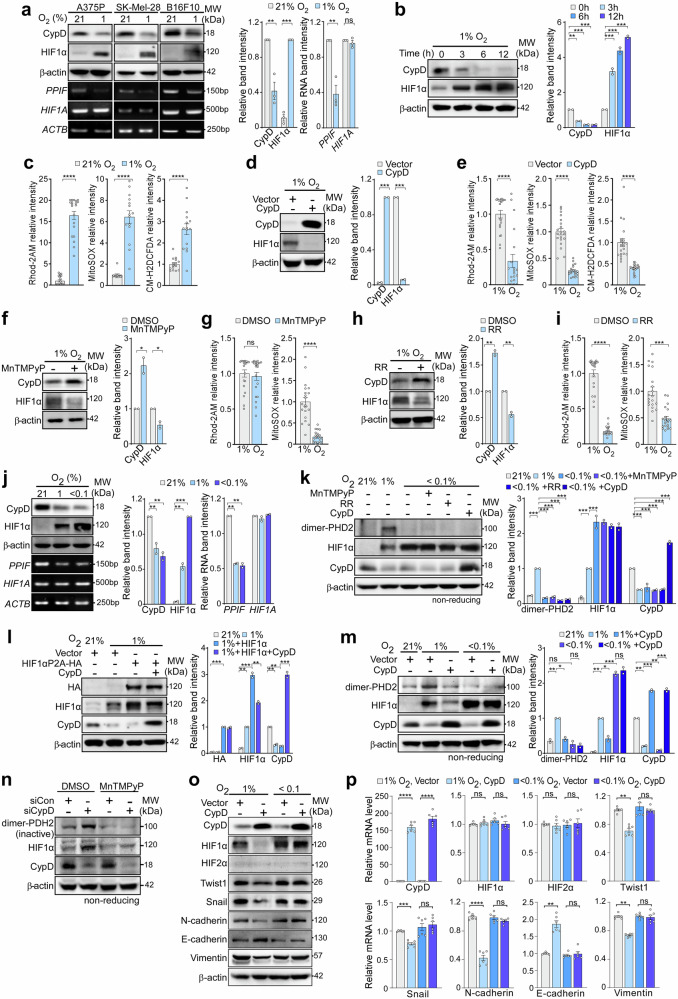
Reciprocal regulation of CypD and HIF1α in mild hypoxia. **a** Expression of CypD and HIF1α under normoxic (21% O_2_) or mild hypoxic (1% O_2_) conditions. Cells were incubated for 24 h in an atmosphere containing 21% or 1% oxygen, and then analyzed by western blotting (upper) and RT-PCR (bottom). **b** HIF1α and CypD expression following mild hypoxic exposure. A375P cells were incubated in 1% O_2_ for the indicated time, and then analyzed by western blotting. **c** Mitochondrial calcium and ROS levels under mild hypoxia. A375P cells were incubated under 21% or 1% O_2_ for 24 h, and then stained with Rhod-2AM, MitoSOX, or CM-H2DCFDA. Subsequently, the cells were analyzed by confocal or fluorescence microscopy and quantified (n = 20). **d** Expression of HIF1α upon overexpression of CypD under mild hypoxia. A375P cells in 1% O_2_ were transfected with pcDNA (Vector) and pcDNA-CypD (CypD) for 24 h, and then analyzed by western blotting. **e** Mitochondrial calcium and ROS levels after overexpression of CypD under mild hypoxia. A375P cells in 1% O_2_ were transfected with pcDNA (Vector) and pcDNA-CypD (CypD) were analyzed as in (**c**) (n = 20). **f** HIF1α expression after MnTMPyP treatment under mild hypoxia. A375P cells were cultured with either DMSO or MnTMPyP under mildly hypoxic conditions for 24 h and then analyzed by western blotting. **g** Mitochondrial calcium and ROS levels after MnTMPyP treatment under hypoxic conditions. A375P cells were cultured with either DMSO or MnTMPyP under mild hypoxia, and then stained with Rhod-2AM and MitoSOX. Subsequently, the cells were analyzed by fluorescence microscopy and the signals quantified (n = 20). **h** HIF1α expression after treatment with a mitochondrial calcium uniporter inhibitor (Ruthenium red, RR) under mild hypoxia. A375P cells incubated with either DMSO or 20 μM RR for 24 h were analyzed as in (**f**). **i** Mitochondrial calcium and ROS level after RR treatment in mild hypoxia. A375P cells cultured with either DMSO or RR were analyzed as in (**g**) (n = 20). **j** Expression of CypD and HIF1α under normoxic, mildly hypoxic, or severely hypoxic (<0.1% O_2_) conditions. Cells were incubated as indicated for 24 h, and then analyzed by western blotting (upper) and RT-PCR (bottom). **k** PHD2 dimerization. A375P cells were transfected with pcDNA-CypD (CypD) or treated with RR and MnTMPyP. The cells were analyzed by western blotting under non-reducing conditions. **l** Effect of CypD on the stability of HIF1α P2A. CypD and HIF1α P2A with HA tag (HIF1α P2A-HA) were overexpressed in A375P cells under mild hypoxia, and the cells were analyzed by western blotting. **m** PHD2 dimerization following overexpression of CypD under severe hypoxia. A375P cells were transfected and incubated as indicated, and then analyzed by western blotting under non-reducing conditions. **n** PHD2 dimerization following CypD silencing and treatment with MnTMPyP. A375P cells transfected with control or CypD siRNA were incubated with MnTMPyP for 24 h, and analyzed by western blotting under non-reducing conditions. **o** Expression of EMT and HIF1α after overexpression of CypD under mild or severe hypoxia. A375P cells were transfected with vector or CypD, cultured as indicated for 24 h, and then analyzed by western blotting. **p** Analysis of relative mRNA abundance after overexpression of CypD under mild or severe hypoxia. A375P cells collected in (**o**) were analyzed by RT-qPCR (n = 6). For figures containing western blot and RT-PCR results, quantification of band intensity was performed using ImageJ and presented on the right. Data are presented as the mean ± SEM. **, *p* < 0.01; ***, *p* < 0.001; ****, *p* < 0.0001; ns not significant

To investigate the potential mutual regulatory interactions between HIF1 activation and CypD suppression, we exposed melanoma cells to mild hypoxia (1% O_2_), which not only resulted in HIF1α stabilization but also led to reduced expression of CypD at both the protein and mRNA levels (Fig. 4a). Expression of CypD and HIF1α showed a strong inverse correlation immediately after induction of hypoxia (Fig. 4b). The hypoxia-induced reduction in CypD closed the mPTP, considering increased mitochondrial calcium accumulation, and subsequently elevated mitochondrial and cellular ROS levels (Fig. 4c). This indicates that activation of HIF1α under conditions of mild hypoxia reduces CypD expression in melanoma cells and subsequently increases ROS levels, the factor essential for pseudohypoxic HIF1α stabilization.

Surprisingly, CypD OE was sufficient to inhibit stabilization of the HIF1α protein under conditions of mild hypoxia (Fig. 4d), suggesting that pseudohypoxic conditioning in response to CypD inhibition is required for HIF1α stabilization under these conditions. Consistent with this, we found that overexpression of CypD led to opening of the mPTP, as evidenced by reduced mitochondrial calcium levels, and a reduction in mitochondrial/cellular ROS levels (Fig. 4e). Furthermore, similar to CypD inhibition-induced pseudohypoxia, stabilization of HIF1α under mild hypoxia was fully reversed by inhibiting mitochondrial ROS production and lowering mitochondrial calcium levels by treatment with MnTMPyP and RR, respectively (Fig. 4f–i). Collectively, these data indicate that ROS, rather than oxygen concentration, is the factor critical for HIF1α stabilization under conditions of mild hypoxia (similar to its role under pseudohypoxic conditions, which requires mPTP closure through CypD suppression).

### Regulation of pseudohypoxia is necessary to activate HIF1α under conditions of mild, but not severe, hypoxia

Expression of CypD decreased under conditions of severe hypoxia (oxygen concentration <0.1%) in a manner similar to mildly hypoxic conditions (Fig. 4j); however, treatments that blocked the pseudohypoxic state under conditions of mild hypoxia, i.e., CypD OE, reducing mitochondrial calcium (RR), and scavenging mitochondrial ROS (MnTMPyP), never affected HIF1α stabilization under severe hypoxic conditions (Fig. 4k). Therefore, in contrast to mild hypoxia, the data suggest that HIF1α stabilization under severe hypoxia is independent of ROS.

Both mitochondrial ROS and oxygen deficiency can inhibit the enzyme activity of HIF prolyl hydroxylases (PHDs), thereby increasing HIF1α stability.^36–38^ Thus, we speculated about the different mechanisms that regulate PHDs under conditions of mild and severe hypoxia. The stability of HIF1α P2A, which lacks hydroxylation sites for PHDs,^23,24^ was not affected by CypD OE under conditions of mild hypoxia (Fig. 4l), confirming PHD-mediated regulation of HIF1α. Among the different PHD isoforms, inhibition of PHD2, but not PHD1 and PHD3, by siRNA increased HIF1α protein stability, as previously reported,^39,40^ and reduced expression of CypD (Supplementary Fig. 6a, b). Under mildly hypoxic conditions, formation of oxidatively homodimerized inactive PHD2 increased markedly, and was reversed by CypD OE; however, under severe hypoxia, there was no increase in the inactive PHD2 dimer (Fig. 4k, m). Similarly, CypD KD under normoxic conditions increased PHD2 dimerization, which was reversed by treatment with a mitochondrial ROS scavenger (Fig. 4n; Supplementary Fig. 6c). These data indicate that establishment of pseudohypoxia in melanoma cells under mildly hypoxic and normoxic conditions is caused by oxidative inactivation of PHD2, whereas severe hypoxia suppresses PDH2 activity due to lack of oxygen. Consistent with these findings, CypD OE did not affect HIF1α stability or expression of EMT feature genes under severely hypoxic conditions (Fig. 4o, p**;** Supplementary Fig. 6d).

Taken together, the data suggest that the mechanism that regulates HIF1α differs under conditions of mild and severe hypoxia; under mild hypoxia, PHD is inactivated by increased ROS production, whereas under severe hypoxia, decreased oxygen levels reduce PHD enzyme activity.

### HIF1α negatively regulates CypD through miR-23a/27a

**Fig. 5:**
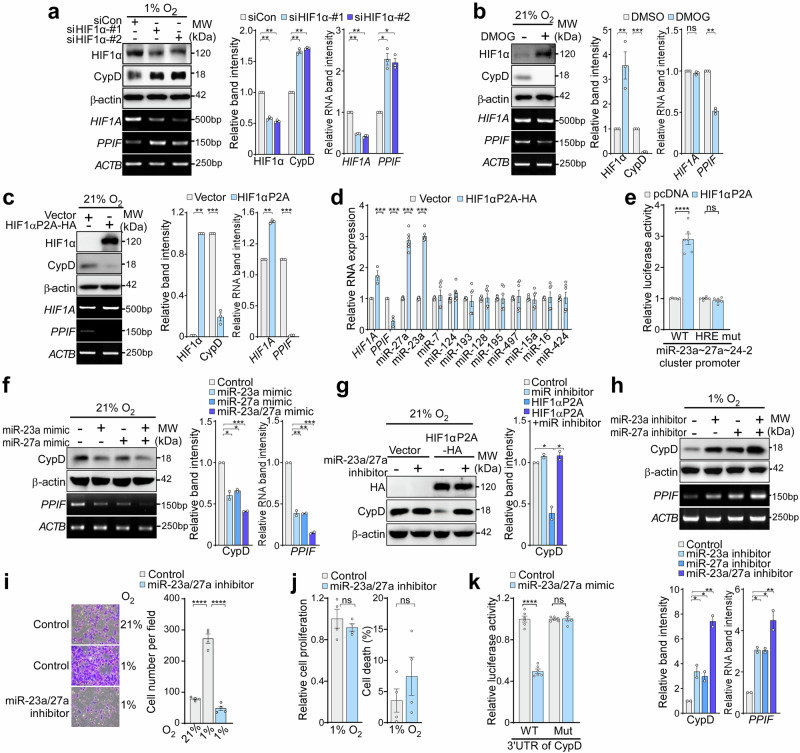
Negative regulation of HIF1α in CypD expression through miR23/27a. **a** CypD expression after silencing HIF1α. A375P cells were transfected for 24 h with control siRNA or siRNA targeting HIF1α under 1% O_2,_ and then analyzed by western blotting and RT-PCR. **b** Expression of CypD after DMOG treatment. A375P cells were treated with DMSO or DMOG, and then analyzed by western blotting and RT-PCR. **c** Expression of CypD after overexpression of HIF1α P2A. A375P cells were transfected with pcDNA (Vector) or pcDNA-HIF1α P2A-HA plasmids, and then analyzed by western blotting and RT-PCR. **d** Expression of 11 candidate miRNAs predicted to target CypD (*PPIF gene)*. A375P cells were treated as in (**c**), and the expression of 11 miRNAs in A375P cells was analyzed by RT-qPCR. **e** Interaction between HIF1α and the miR-23a ~ 27a ~ 24-2 cluster promoter. A375P cells were co-transfected with luciferase reporter plasmids containing either the wild-type (WT) or HRE-mutated (HRE mut) miR-23a ~ 27a ~ 24-2 promoter, along with either pcDNA or pcDNA-HIF1α P2A constructs. Luciferase activity was measured and compared between groups. **f** CypD expression after treatment with a miRNA mimic under normoxia. A375P cells were transfected for 24 h with miR-23a and miR-27a mimics as indicated, and then analyzed by western blotting. **g** Effect of miRNA inhibitors on CypD expression after overexpression of HIF1α P2A. A375P cells were transfected with pcDNA-HIF1α P2A-HA plasmid and treated with inhibitors of miR-23a and miR-27a for 24 h. The cells were analyzed by western blotting. **h** Expression of CypD after treatment with miRNA inhibitors under mild hypoxia. A375P cells transfected with inhibitors of miR-23a and miR-27a under 1% O_2_ were analyzed by western blotting. **i** Cell invasion after miR23a/27a inhibition. The invasive capacity of A375P cells was assessed using Transwell invasion assay for 24 h following treatment with Control or miR-23a/27a inhibitor. Scale bar, 50 µm. The number of invaded cells was quantified (n = 4). **j** Cell proliferation and cell death after miR23a/27a inhibition under hypoxia. A375P cells were treated with miR23a/27a inhibitor, and cell viability was assessed using an MTT assay. **k** Luciferase assay for miRNA-mRNA interaction. A375P cells transfected with psiCHECK-2 constructs containing either the wild-type 3′UTR of *PPIF* (WT) or a 3′UTR with a mutated miR-23a/27a binding site. Cells were then treated with miRNA mimic, and luciferase activity was measured. For figures containing western blot and RT-PCR results, quantification of band intensity was performed using ImageJ. Data are presented as the mean ± SEM. **, *p* < 0.01; ***, *p* < 0.001; ****, *p* < 0.0001; ns not significant

To further investigate reciprocal regulation between HIF1α on CypD expression, HIF1α under mild hypoxia was inhibited by siRNAs. Inhibiting HIF1α resulted in a significant increase in both CypD mRNA and protein levels (Fig. 5a). Conversely, forced stabilization of HIF1α by treatment with DMOG, an inhibitor of PHD, or OE of HIF1α P2A led to reduced expression of CypD at both the mRNA and protein levels under normoxic conditions (Fig. 5b, c). These data indicate that HIF1α negatively regulates the expression of CypD at the transcriptional level.

Since we did not identify a putative HIF1 binding site in the CypD promoter (from −1 kb to the transcription start) using PROMO prediction analysis (http://alggen.lsi.upc.es/), we next explored the potential negative regulation of CypD through miRNAs. TargetScan analysis (https://www.targetscan.org) identified 11 miRNAs as potential regulators targeting CypD. Among those miRNAs, miR-23a and miR-27a were upregulated by more than 2-fold upon HIF1α P2A OE in cancer cells (Fig. 5d). miR-23a and miR-27a are transcribed as a part of the miR-23a ~ 27a ~ 24-2 cluster, and are elevated in various cancer cells.^41,42^ Using a luciferase reporter assay containing the miRNA cluster promoter, we found that HIF1α P2A OE significantly increased luciferase activity in the original promoter sequence but not in the mutated sequence with a defective hypoxia response element (HRE) (Fig. 5e), confirming that HIF1α directly binds to and transcriptionally activates the miRNA cluster promoter. Treatment of miR-23a or miR-27a mimics significantly reduced CypD expression under normoxic conditions (Fig. 5f). Conversely, miR-23a or miR-27a inhibitors rescued CypD expression following its suppression by HIF1α P2A OE (Fig. 5g). Similarly, under hypoxic conditions, treatment with miRNA inhibitors increased CypD expression and completely suppressed melanoma cell invasiveness (Fig. 5h, i), while having no significant effect on cell proliferation or cell death (Fig. 5j). To further validate the direct interaction between miR-23a/27a and CypD mRNA, we performed a dual-luciferase reporter assay using the CypD 3′UTR, which contains the predicted miRNA binding sites (Fig. 5k). Treatment of miR-23a and miR-27a mimics significantly reduced luciferase activity, indicating that these miRNAs directly target CypD mRNA. Furthermore, introducing mutations in the predicted miR-23a/27a binding sites within the 3′UTR completely abolished this inhibitory effect, demonstrating that CypD suppression occurs through direct miRNA-mRNA binding. Thus, our results show that HIF1α induces expression of miR-23a and miR-27, which suppress CypD expression in melanoma cells, indicating a reciprocal regulatory loop involving HIF1α stabilization and CypD suppression.

### Conservation of the CypD-HIF1α regulatory mechanism in metastatic liver and prostate cancers

To determine whether the mechanism identified in melanoma is applicable to other cancer types, we examined SK-Hep1 and PC3 cells, which originate from hepatocellular carcinoma and prostate cancer, respectively, and are known for their high metastatic properties.^43,44^ Under normoxic conditions, CypD knockdown resulted in increased levels of HIF1α, EMT markers, ROS production, and invasiveness in both cell lines (Supplementary Fig. 7a–c). Exposure to hypoxic conditions led to an increase in HIF1α levels and a consequent decrease in CypD expression, while CypD OE under hypoxia reduced HIF1α levels, EMT marker expression, and invasiveness (Supplementary Fig. 7d–f). In both cell lines, HIF1α P2A OE induced the expression of miR-23a and miR-27a, and treatment with miR-23a/27a mimics suppressed CypD expression (Supplementary Fig. 7g–i). Moreover, hypoxia-induced inhibition of CypD expression was increased by miR-23a/27a inhibitors (Supplementary Fig. 7j). These findings suggest that the CypD-HIF1α regulatory mechanism is conserved in other cancer cells, highlighting its broader relevance beyond melanoma.

### Adenoviral overexpression of CypD in tumors effectively blocks metastasis

**Fig. 6:**
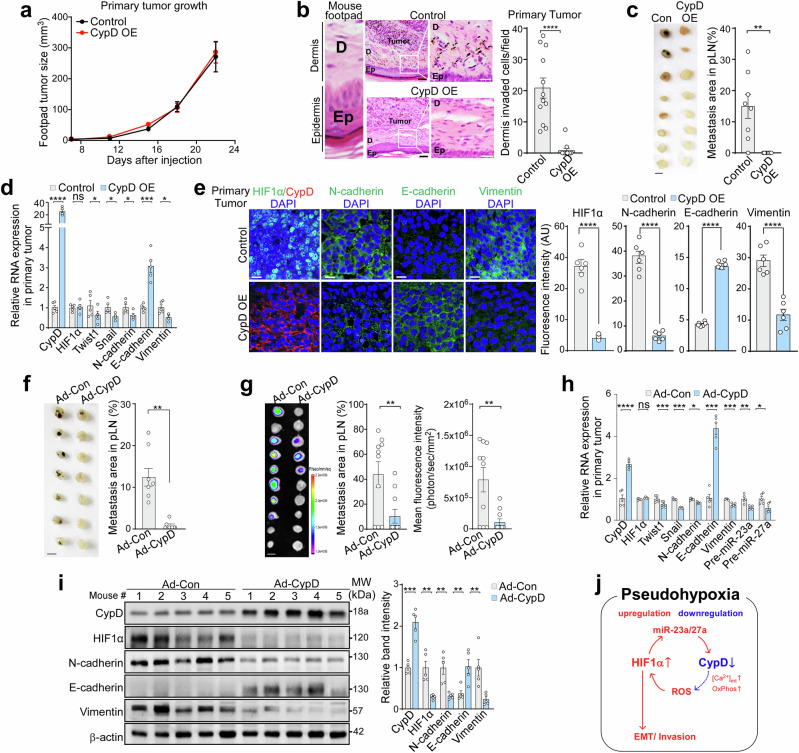
Blocking melanoma metastasis through overexpression of CypD. **a** Primary tumor growth. Control or CypD OE B16F10 cells were injected subcutaneously into the footpads of mice (tumor n = 8, 4 mice per group), and tumor volume was measured using a caliper. **b** Invasion into the dermis. The primary tumor tissues collected in (**a**) were stained with H&E (left). The infiltrating melanoma cells (black arrow) from primary tumors were counted (right, n = 12). Black scale bar, 40 µm; white scale bar, 50 µm. **c** Lymph node (LN) metastasis. Popliteal lymph nodes (pLNs) were collected at the end of the experiment in (**a**) (left, lymph node n = 8 per group), and the pigmented metastatic area was calculated using ImageJ (right). Scale bar, 1 mm. **d** Gene expression analysis. Total RNA from tumor tissues collected from (**a**) was analyzed by RT-qPCR. Relative mRNA levels of CypD OE tumors were compared with those in control tumors (n = 5). **e** Expression of HIF1α, CypD and EMT markers in primary tumors. Primary tumors collected from the mice in (**a**) were analyzed by immunohistochemistry (left). Quantification of fluorescence intensity (right, n = 6). Scale bar, 10 µm. Inhibition of lymph node metastasis by Ad-CypD. B16F10 (**f**) and A375P cells (**g**) were injected subcutaneously into the footpads of mice. After one week, either a control (Ad-Conn) or a CypD-overexpressing adenovirus (Ad-CypD) was administered intratumorally. pLNs were isolated and analyzed as in (**c**), 24 days (**f**), and 40 days (**g**) after virus injection (**f**, n = 7 per group; **g**, n = 10 per group). Scale bar, 1 mm. **h**, **i** RNA and protein expression. Tissues isolated from the A375P tumors shown in (**g**) were analyzed by RT-qPCR (**h**, n = 5) and western blotting (**i**, n = 5). **j** Reciprocal regulatory loop between HIF1α stabilization and CypD suppression to establish pseudohypoxic conditions. Data are presented as the mean ± SEM. *, *p* < 0.05; **, *p* < 0.01; ***, *p* < 0.001; ****, *p* < 0.0001; ns not significant

To investigate the importance of pseudohypoxic regulation on metastasis in vivo, control or CypD OE B16F10 cells were allografted into the footpads of C57BL/6 mice, and primary tumor growth and popliteal lymph node metastasis were analyzed. While the growth of the primary tumor remained unremarkable (Fig. 6a), CypD OE reduced infiltration of cancer cells into the footpad epidermis significantly (Fig. 6b), as well as metastasis to the popliteal lymph nodes (Fig. 6c), compared with controls. Additionally, CypD OE reduced expression of hallmark EMT markers at the mRNA and protein levels without impacting HIF1α mRNA expression in the primary tumor (Fig. 6d, e). The anti-metastatic effect of CypD OE was fully reversed by forced HIF1α P2A overexpression, restoring EMT features and metastatic potential (Supplementary Fig. 8a–c). Taken together, these findings indicate that CypD OE in cancer cells impedes the HIF1α-EMT pathway in vivo, resulting in effective suppression of metastasis while leaving growth of the primary tumor unaffected.

Therefore, to examine the potential therapeutic application of CypD OE, we generated an adenovirus overexpressing CypD (Ad-CypD) and administered it to B16F10 allografts grown in the footpads of C57BL/6 mice and A375P xenografts in the footpads of nude mice. In both models, injection of Ad-CypD significantly reduced metastasis to the lymph nodes but did not affect primary tumor growth (Fig. 6f, g; Supplementary Fig. 9a, b). Analysis of primary tumor samples from Ad-CypD-treated mice revealed a reduction in EMT marker mRNAs and miR-23a/27a (Fig. 6h; Supplementary Fig. 9c). Consistently, protein levels of HIF1α, N-cadherin, and vimentin decreased, while E-cadherin levels increased (Fig. 6i; Supplementary Fig. 9d). Similarly, in an orthotopic xenograft model of human A375P tumors implanted in the footpads of NOD/SCID/IL2rγ^null^ (NSG) mice, intratumoral injection of Ad-CypD completely blocked distant metastasis to the liver and lungs, accompanied by the suppression of EMT-related gene expression (Supplementary Fig. 10a–d). In addition, to assess the effect of ROS scavenging on metastasis, we administered NAC via drinking water to B16F10 allograft mice. Although NAC had no effect on primary tumor growth (Supplementary Fig. 11a), it significantly inhibited lymph node metastasis, which was associated with disruption of pseudohypoxic signaling, as indicated by elevated CypD expression, decreased HIF1α expression, and reduced EMT features (Supplementary Fig. 11b–d). Moreover, in the NAC-treated group, angiogenesis and macrophage infiltration were both reduced, as evidenced by decreased VEGF, CD31, and F4/80 staining in primary tumor tissues (Supplementary Fig. 12a–c). Notably, similar but more pronounced reductions were observed in tumors overexpressing CypD via adenoviral delivery (Supplementary Fig. 12d–f), indicating that CypD–HIF1α signaling may play multifaceted roles in tumor progression.

Collectively, these data strongly indicate that the pseudohypoxic conditions generated by the reciprocal regulatory loop between HIF1α and CypD are crucial for cancer metastasis. Inactivating one of the components of this loop, for example, through CypD OE, is sufficient to exert potent inhibition of HIF1 and subsequent blockade of metastasis.

## Discussion

In this study, we discovered that melanoma cells under mild hypoxia establish a pseudohypoxic state by raising ROS levels, thereby stabilizing HIF1α in an oxygen-independent manner. This process requires sequential mitochondrial events: inhibition of CypD to close the mPTP, increased calcium accumulation to increase OxPhos, and subsequent ROS elevation to inhibit PHD. Consequently, activated HIF1α suppresses CypD by inducing expression of miR-23a/27a, thereby creating a feedback regulatory loop to support HIF1α stabilization and CypD suppression. This mechanism is essential for sustaining HIF1 signaling under fluctuating oxygen levels, ranging from mild hypoxia to normoxia, during melanoma metastasis. Notably, CypD OE was sufficient to disrupt the pseudohypoxic state and trigger HIF1α degradation, thereby inhibiting metastasis. These findings underscore, for the first time, that the mitochondrial ROS production mediated by mPTP regulation, rather than oxygen levels in the TME, is crucial for HIF1 signaling and melanoma metastasis.

Melanoma does not originate from epithelial tissues and therefore frequently expresses some lineage-specific factors associated with EMT, which contribute to its high metastatic propensity.^45,46^ However, the initiation of metastasis in melanoma involves extensive alterations in numerous molecular pathways, including those regulating cell adhesion, migration, and invasion, necessitating additional gene expression regulation beyond lineage-specific factors.^47^ Additionally, phenotypic transitions between highly metastatic cells with mesenchymal-like signatures and less metastatic cells with epithelial-like signatures have been observed in melanoma.^46,48^ These transitions highlight the phenotypic plasticity of melanoma cells and underscore the relevance of studying EMT in this non-epithelial cancer. Furthermore, EMT also plays a crucial role in tumorigenesis by promoting angiogenesis, evading immune responses, increasing adaptability, and consequently enhancing resistance to various therapies in melanoma.^49–51^ Therefore, the pseudohypoxic mechanism identified in this study could be exploited to not only restrict the metastatic dissemination of melanoma cells but also regulate their tumorigenic potential and improve therapeutic outcomes. However, considering that metastatic cells at distant sites often undergo mesenchymal–epithelial transition (MET), further studies are needed to determine how the CypD–HIF1α circuit is regulated throughout the multi-stage metastatic process.

The interplay between mitochondrial function and HIF1 signaling has been reported in various contexts, including the production of metabolites such as ROS and succinate, and transcriptional regulation of metabolic gene expression.^52^ In the context of mitochondrial respiration, some studies show that inhibiting mitochondrial respiration destabilizes HIF1α via PHD activation. The mechanism was attributed largely to decreased cellular oxygen levels, which fell below the necessary threshold for PHD activity due to excessive consumption of available oxygen by mitochondrial respiration^53–55^; however, our study found that this mechanism was not operational, at least in melanoma cells. Meanwhile, some reports are consistent with our own, showing that increased OxPhos increases ROS production, leading to the hypoxic stabilization of HIF1α.^36,56,57^ These studies specifically demonstrate that ROS, rather than oxygen depletion, is the primary factor responsible for PHD inhibition.^36,37,58^ Although the studies do not differentiate between mild and severe hypoxia, all of the data were obtained under conditions of mild hypoxia (oxygen concentrations of 1% or higher). As such, the oxygen-dependent mechanisms that regulate HIF1α remain controversial, likely due to differences in cellular context, and may vary depending on the cellular oxygen concentration (mild versus severe hypoxia); however, at least in melanoma, ROS production resulting from elevated OxPhos activity after mPTP closure is a major mechanism that regulates HIF1α and subsequent metastatic progression.

In addition to our findings of reduced CypD expression in metastatic cancer patients, it was reported that the average oxygen levels in primary tumor tissues often exceed 1% in many cancers, including melanoma,^59–61^, suggesting that cancer cells are typically exposed to mild rather than extreme hypoxia. These findings underscore the clinical relevance and significance of the mild hypoxic HIF1α regulation demonstrated in this study. Furthermore, as metastasis progresses, cancer cells migrate toward oxygen-rich vasculature, likely exposing them to oxygen concentrations of 1% or higher. Consequently, cancer cells undergoing metastasis cannot rely solely on oxygen-dependent regulation of PHD to maintain HIF1α stability. Instead, the self-reinforcing pseudohypoxic induction mediated by mPTP, independent of environmental oxygen levels, is crucial for migrating cancer cells, given the necessity of sustained HIF1 signals for metastasis.^62^

Specific HIF2α inhibitors, such as PT2385 and its derivatives, which target the PAS-B domain of HIF2α and block its interaction with HIF1β, have been developed and are currently under undergoing clinical trials.^63,64^ In contrast, although many small-molecule HIF1α inhibitors have been developed, these compounds do not directly bind to HIF1α.^65^ For instance, widely used HIF1α inhibitors such as topotecan and PX-478 act by inhibiting the transcription or translation of HIF1α rather than directly targeting the protein.^66,67^ Notably, the anticancer effects of topotecan in clinical use are primarily attributed to its general cytotoxic properties triggered by damaging DNA rather than its impact on HIF1α. Thus, there remains a need to improve the specificity and efficacy of current HIF1α inhibitors.^65^ In addition, peptide and RNA antagonists have been developed; however, these approaches are still in their early stages.^68,69^ Therefore, the novel HIF1α inhibition strategy, utilizing CypD OE and ROS scavengers, holds promise for potent HIF1 inhibition and metastasis suppression with relatively low toxicity. Combining this strategy with existing HIF1α inhibitors and chemotherapies could be an effective anticancer approach for treating aggressive metastatic cancers.

In addition to its direct role in promoting EMT and metastasis, CypD inhibition and subsequent HIF1α activation profoundly affect the TME. Consistently, we observed upregulated expression of TME-associated genes involved in inflammation, angiogenesis, and extracellular matrix (ECM) remodeling, all of which contribute to metastasis by modulating stromal cell activity and altering the ECM.^70,71^ Given the crucial role of TME in tumor progression, our findings suggest that the pseudohypoxic state induced by CypD inhibition not only enhances the intrinsic metastatic potential of cancer cells but also reprograms the TME, creating a more permissive environment for tumor dissemination. Thus, targeting the pseudohypoxic mechanism, either through CypD OE or antioxidant treatment, could not only suppress cancer cell metastasis but also shift the TME toward a less tumor-supportive state.

Cytochrome *c* oxidase (COX), also known as Complex IV, delivers electrons to molecular oxygen; thus, similar to PHD, the function of COX is thought to depend on the oxygen concentration. However, mild hypoxia alone cannot affect COX enzyme activity, since the oxygen concentration required for half-maximal respiration (*K*_m_) of COX is estimated to be less than 1 μM, which is lower than the oxygen concentration under conditions of mild hypoxia (~10 μM oxygen).^61,72,73^ Consistent with this, oxygen concentrations did not affect mitochondrial respiration significantly until they dropped to levels consistent with severe hypoxia (~0.1%).^74^ Furthermore, HIF1 activation caused by mild hypoxia induces gene expression, leading to switching of the subunit composition of the multiprotein complex COX to improve mitochondrial respiration and facilitate hypoxic adaptation.^75^ Thus, COX might have evolved a low *K*_m_ value to ensure uninterrupted respiration and ATP production, even in the presence of reduced mitochondrial oxygen levels due to high respiration (oxygen consumption). We believe that melanoma cells exploit this crucial property of COX not only to maintain consistent cellular energy levels under changing oxygen concentrations but also to sustain metastatic competence by establishing pseudohypoxic conditions. Thus, pseudohypoxic induction should not be viewed as an accidental “passive” state resulting from an imbalance of mitochondrial functional due to low oxygen levels, but rather as an “active” pro-tumorigenic mechanism triggered by the HIF1 pathway; this state is essential for metastasis.

Unlike other cancers, melanoma lesions are both visible and easily accessible on the skin surface, allowing for straightforward intratumoral administration. In particular, certain subtypes of melanoma exhibit distinct patterns of regional metastasis, including in-transit metastases, where cancer cells spread through lymphatic vessels between the primary tumor and regional lymph nodes. While surgery may be effective for isolated lesions, extensive disease burden or the presence of multiple lesions often limits the utility of surgical resection alone due to a high risk of recurrence.^76^ To address this, intratumoral injection of the adenovirus overexpressing CypD (Ad-CypD) into accessible lesions could serve as a practical and localized therapeutic strategy to suppress further metastatic progression. Under this metastasis-suppressed condition, combining CypD-based local therapy with immune checkpoint inhibitors or conventional cytotoxic anticancer drugs, already used in clinical melanoma treatment, may enhance overall therapeutic efficacy. Alternatively, oral antioxidant therapies such as NAC could be explored as a systemic approach to further enhance treatment outcomes in melanoma and potentially extend to other solid tumors exhibiting pseudohypoxic mechanisms, aiming to disrupt these mechanisms and inhibit their metastatic potential.

To summarize, we have identified a mechanism responsible for mitochondria-mediated induction of pseudohypoxia that is essential for HIF1α stabilization and melanoma metastasis. Inhibiting these pseudohypoxic conditions effectively blocks HIF1 signaling and subsequent metastasis, offering a novel therapeutic strategy for treating metastatic cancers.

## Materials and methods

### Cell culture and quantification of cellular ROS and mitochondrial calcium levels

Human skin (A375P and SK-Mel-28) and mouse skin (B16F10) cancer cells were purchased from the American Type Culture Collection (ATCC, USA) and cultured as recommended by the supplier in DMEM medium (GIBCO, USA) containing 10% fetal bovine serum (FBS; GIBCO, USA) and 1% penicillin/streptomycin (GIBCO, USA), and at 37 °C in a humidified atmosphere of 5% CO_2_. For hypoxic culture, cells were cultured in a hypoxic chamber (Astec, Japan) or using oxygen absorbers and oxygen indicators (MGCC, Japan). To measure cellular and mitochondrial ROS, cells were incubated for 30 min with 10 μM CM-H2DCFDA (Invitrogen, USA) or 5 μM MitoSOX (Invitrogen, USA), respectively, and analyzed by flow cytometry. To measure mitochondrial calcium, cells were incubated for 30 min at 37 °C in Krebs-Ringer-HEPES (KRH) buffer containing 10 μM Rhod-2AM (Invitrogen, USA), and then analyzed by confocal microscopy. After background subtraction, the fluorescence signal was quantified as the average pixel value within the selected region of interest. The fold change was calculated based on the average pixel value of the control group.

### Plasmid, antibodies, and chemicals

HA-HIF1αlpha P402A/P564A-HA-pcDNA3 was a gift from William Kaelin (Addgene plasmid #18955). Anti-HIF2α and anti-vimentin were purchased from Cell Signaling Technology; an anti-CypD antibody was purchased from Thermo Fisher Scientific; anti-Twist, anti-Snail antibodies were purchased from ABclonal Technology; anti-E-cadherin and anti-N-cadherin antibodies were purchased from BD Biosciences; an anti-HIF1α antibody was purchased from Abcam; and an anti-β-actin antibody was purchased from MP Biomedicals. All chemicals were purchased from Sigma unless stated otherwise.

### Generation of CypD knockout (*Ppif*^*−/−*^) or overexpressing stable cells

B16F10 cells in which CypD is knocked out were generated using clustered regularly interspaced short palindromic repeats (CRISPR)/CRISPR-associated protein 9 (Cas9) gene editing technology.^77^ The guide RNA (gRNA) expression vector and the pRGEN-Cas9 expression vector were transfected into B16F10 using Lipofectamine (ToolGen, Inc., Korea). Two days after transfection, cells were cultured in DMEM containing 1 μg/mL puromycin, and stably transfected clones were selected. To verify the phenotype of the surviving clones, genomic DNA was isolated, and deletion of *Ppif* confirmed in a T7E1 assay. Lack of CypD expression was confirmed by western blotting. The gRNA for *Ppif*, designed by ToolGen, Inc. (Republic of Korea), has the following sequence: 5′-CGCTCGTGTACTTGGACGTGG-3′ (*Ppif*). To generate cell lines showing stable expression of CypD, B16F10 cells were transfected with pcDNA or pcDNA-CypD (*Ppif)* and cultured in DMEM containing 2 mg/mL G418. Resistant clones were selected and overexpression of CypD was confirmed by western blot analysis of cell lysates. To generate A375P cells stably expressing GFP, lentiviral transduction was performed using lentiviral particles carrying the GFP gene. GFP-positive cells were sorted using a BD FACSAria™ III Cell Sorter (BD Biosciences, USA) and subsequently expanded in culture.

### Cell proliferation and invasion assays

To assess cell proliferation, cells were serially diluted (1:2) in 96-well plates and cultured for 24 or 48 h. The cells were incubated with MTT reagent at 37 °C for 3 h. The medium was then discarded and the cells resuspended in 200 μL of DMSO. Absorbance was measured at OD_590_ nm and the relative cell proliferation rate was calculated. Invasion assays were performed using a Transwell system (8-μm pore size, Falcon, USA). Briefly, 5 × 10^4^ cells were seeded onto the apical side of a Transwell chamber (24-well insert) pre-coated with Matrigel (BD Biosciences, USA) and then cultured under serum starvation conditions. Conditioned medium from NIH3T3 fibroblast cells was added to the basal compartment as a chemoattractant. After 24 h, the cells remaining on the apical side of the chamber were removed, and the cells that had migrated to the basal surface were fixed for 10 min in methanol, stained with 0.2% crystal violet or DAPI, and then photographed under a light microscope (Olympus, Japan) at ×200 magnification.

### RNA extraction, reverse transcription PCR (RT-PCR), and real-time PCR (RT-qPCR)

Total RNA, including mRNA and miRNA, was extracted from cancer cells using Trizol reagent (Invitrogen, USA), and cDNA was synthesized using the ProtoScript First Strand cDNA Synthesis Kit (New England Biolabs, USA). For miRNA analysis, cDNA was synthesized using Mir-X TM miRNA First Strand synthesis (TaKaRa Biotechnology Co. Ltd, Japan), followed by TB Green Real-time PCR (TaKaRa Biotechnology Co. Ltd, Japan). RT-PCR was performed in a PCR SimpliAmp Thermal Cycler (Applied Biosystems, USA), or RT-qPCR was performed using the SYBR Premix Ex Taq II kit (TaKaRa Biotechnology Co. Ltd, Japan) and a Light Cycler 480 real-time PCR system (Roche Diagnostics, Germany). The primer sequences used for PCR are provided in Supplementary Table 1.

### TargetScan analysis

To investigate the miRNA-mediated regulation of CypD (*PPIF*), TargetScan (version 8.0; https://www.targetscan.org/) was used to predict putative miRNA target sites in the 3′ untranslated region (UTR) of *PPIF*. The *PPIF* 3′ UTR sequence was analyzed based on sequence conservation and context scores to identify candidate miRNAs with high binding potential. The predicted miRNA-mRNA interactions were further validated through Pearson correlation analysis using experimental gene expression data to prioritize functionally relevant miRNA candidates.

### Treatment of siRNA, miRNA mimics, and miRNA inhibitors

Briefly, siRNAs, miRNA mimics, and miRNA inhibitors were synthesized by Genolution (Korea); the sequences are as follows: human CypD siRNA -#1, 5′-GGCAGAUGUCGUCCCAAAG-3′; human CypD siRNA-#2, 5′-GAUAAGGGCUUCGGCUACA-3′; mouse CypD siRNA-#1, 5′-GCAGAUGUCGUGCCAAA-3′; mouse CypD siRNA-#2, 5′- GAGAAGGGCUUUGGC-UACA-3′; human HIF1α-siRNA-#1, 5′-UGUGAGUUCGCAUCUUGAU-3′; human and mouse HIF1α siRNA-#2, 5′-GCCACUUCGAAGUAGUGCU-3′; mouse HIF1α siRNA-#1, 5′- UGUGAGCUCACAUCUUGAU-3′; control siRNA, 5′-ACUCUAUCUGCACGCUGAC-3′; miR-23a mimic, 5′- GGGGUUCCUGGGGAUGGGA-UUU-3′; miR-23a inhibitor, 5′-GCGGAACUUAGCCACUGUGAA-3′; miR-27a mimic, 5′-UUCACAGUGGCUAAGUU-CCGC-3′; miR-27a inhibitor, 5′-GCGGAACUUAGCCACUGUGAA-3′; miRNA mimic (control), 5′-UUCUCCGAACGUGUCACGUTT-3′; and miRNA inhibitor (control), 5′-CAGU-ACUUUUGUGUAGUACAA-3′. Cells were cultured to 50–75% confluency in 6-well plates, incubated with 40 nM siRNA or 100 nM miRNA mimics/inhibitors mixed with G-Fectin (Genolution, Korea), and then treated with drugs as indicated.

### Luciferase reporter assay

To verify the binding of miR-23a/27a to *PPIF* mRNA, the 3′UTR sequence of human *PPIF* and its mutated 3′UTR version with disrupted miR-23a/27a binding sites were synthesized by Gene Universal (Nanjing, China). These sequences were cloned into the psiCHECK-2 luciferase reporter vector. A375P cells transfected with the cloned psiCHECK-2 constructs were treated with miR-23a/27a mimics or a miRNA control. After 24 h, the cells were harvested, and luciferase activity was measured using a Promega luciferase assay kit (Promega, Madison, USA). To evaluate the direct binding of HIF1α to the miR-23a ~ 27a ~ 24-2 cluster promoter, 300-bp promoter fragments containing either the wild-type HRE (WT) or a mutated HRE were cloned into the pGL3.0 luciferase reporter plasmid (Promega, Madison, USA). The cloned plasmids were then co-transfected into A375P cells along with either pcDNA-HIF1α P2A or an empty pcDNA vector. After 24 h, the cells were harvested, and luciferase activity was measured and normalized to the total protein concentration.

### Tumor allograft, xenograft, and metastasis models in mice

Animal tumor allograft and xenograft experiments were approved by the UNIST (UNISTIACUC-16-27, UNISTIACUC-20-02). Control or CypD-overexpressing B16F10 cells (100 μL, 2 × 10^5^ cells) were injected subcutaneously into the footpads of C57BL/6 mice. To assess the metastasis-inhibiting effect of CypD overexpression, B16F10 mouse melanoma cells or GFP-expressing A375P human melanoma cells were injected into the footpad of C57BL/6, nude, or NOD/SCID/IL2rγ^null^ (NSG) mice. Once tumors were established, CypD-overexpressing adenovirus or control virus was injected. Tumors were measured twice a week with a caliper, and tumor volume was calculated using the formula V = 1/2 × (width)^2^ × length. At the end of the experiment, the animals were euthanized, and popliteal lymph nodes and tumors were collected. The harvested organ specimens were fixed in 10% formalin and embedded in paraffin for histological analyses. Sections (5-μm thick) were placed on high-adhesive glass slides and analyzed by hematoxylin and eosin (H&E) staining. To confirm the metastatic potential of A375P-GFP cells, GFP fluorescence signals in isolated lymph nodes, lungs, and liver were analyzed using in vivo Xtreme bioimaging equipment (Bruker, Germany). To establish a lung metastasis mouse model, wild-type (WT), CypD knockout (KO), pcDNA stable (Control), or CypD-overexpressing (OE) B16F10 cells were injected into the tail vein of C57BL/6 mice. Subsequently, we harvested, fixed, sectioned, H&E-stained, and analyzed the lungs. The stained slides were scanned at ×20 magnification using a dot slide system virtual microscope (Olympus, Japan). Immunohistochemical (IHC) staining of tissue sections was performed as described previously.^78^

### Analysis of melanoma patient specimens

Melanoma biospecimens and clinical data were obtained from the Biobank of Chonnam National University Hwasun Hospital and the NCC Bio Bank of the National Cancer Center, South Korea. All experiments involving patient specimens were approved by the Institutional Review Boards (IRBs) of UNIST and the National Cancer Center (NCC) under protocol numbers UNISTIRB-24-065-C and NCC2024-0333, respectively. Frozen biopsy samples were analyzed by RT-PCR, while formalin-fixed tissue sections were stained with H&E. IHC staining of tissue sections was performed as previously described.^78^ The stained slides were scanned at ×20 magnification using a dot slide system virtual microscope (Olympus, Japan).

### RNA-seq and data analysis

RNA-seq analysis was performed by Genome Insight company (https://genomeinsight.net/ko, Current, Inocras). WT and CypD KO B16F10 cells cultured under normoxic conditions for 24 h were harvested and total RNA was extracted using the Illustra™ RNAspin Mini RNA Isolation Kit (GE Healthcare). RNA quality was assessed in a 4200 TapeStation System using an RNA Screen Tape (Agilent Technologies), and RNA was quantified using Qubit (Thermo Fisher Scientific). Libraries of total RNAs were constructed using the KAPA RNA HyperPrep Kit with RiboErase (Roche). High-throughput sequencing was performed as paired-end 150 sequencing runs using NovaSeq 6000 (Illumina). Raw reads were assembled, and low-quality reads were filtered using Cutadapt (version 3.4). Filtered reads were aligned to a reference genome downloaded from Ensembl GRCm39 (Mus musculus) using STAR software^79^ (version 2.7.8a), and gene-level expression for each sample was calculated using the RSEM package^80^ (version 1.3.1). Read counts were normalized to ensure effective library size using the DESeq2 package (version 1.26.0).^81^ A two-sided Wald test was performed using the “DESeq2” package (version 1.26.0) to analyze differential expression under different conditions. Pathway analysis was performed using gene set enrichment analysis^82^ (GSEA), and Hallmark pathways obtained from MsigDB^82,83^ were analyzed.

### GEO data and statistical analyses

Survival data for patients with metastatic melanoma were retrieved from the cBioPortal database (http://www.cbioportal.org).^20,21^ mRNA expression array data from human melanoma patients, which included 40 non-metastatic and 41 metastatic patients, were obtained from the Gene Expression Omnibus database^84,85^ (GSE7553). Statistical analyses were performed using the software program Prism 7.0 (GraphPad, USA). Mean comparisons between two groups were performed using unpaired *t*-tests, and a *p*-value < 0.05 was considered significant.

## Supplementary information

Pseudohypoxic stabilization of HIF1α via cyclophilin D suppression promotes melanoma metastasis

Original immunoblot films

## Data Availability

The RNA sequencing data analyzed in this study have been deposited in NCBI’s Gene Expression Omnibus (GEO) under the accession number GSE299396.
