# Supplementary material for: Pseudohypoxic stabilization of HIF1α via cyclophilin D suppression promotes melanoma metastasis
Source: Signal Transduct Target Ther. 2025 Jul 24;10:231. doi: 10.1038/s41392-025-02314-8 (PMC12287346; doi:10.1038/s41392-025-02314-8)
Supplement: Supplementary file 1 — Pseudohypoxic stabilization of HIF1α via cyclophilin D suppression promotes melanoma metastasis [file 41392_2025_2314_MOESM1_ESM.docx]

Supplementary Materials for

Pseudohypoxic stabilization of HIF1α via cyclophilin D suppression promotes melanoma metastasis

**Authors:** Hye-Kyung Park^1^*, Sung Hu^1^, So Yeon Kim^1^, Sora Yoon^2^, Nam Gu Yoon^1^, Ji Hye Lee^1^, Wonyoung Choi^3,4^, Sun-Young Kong^3,5^, Jong Heon Kim^3,6^, Dougu Nam^1^, and Byoung Heon Kang^1^*

Correspondence to: Hye-Kyung Park ([hkparkgene@unist.ac.kr](mailto:hkparkgene@unist.ac.kr)) and Byoung Heon Kang ([kangbh@unist.ac.kr](mailto:kangbh@unist.ac.kr))

**This file includes:**

Supplementary Fig. S1 to S12 with legends

Supplementary Tables S1 to S2


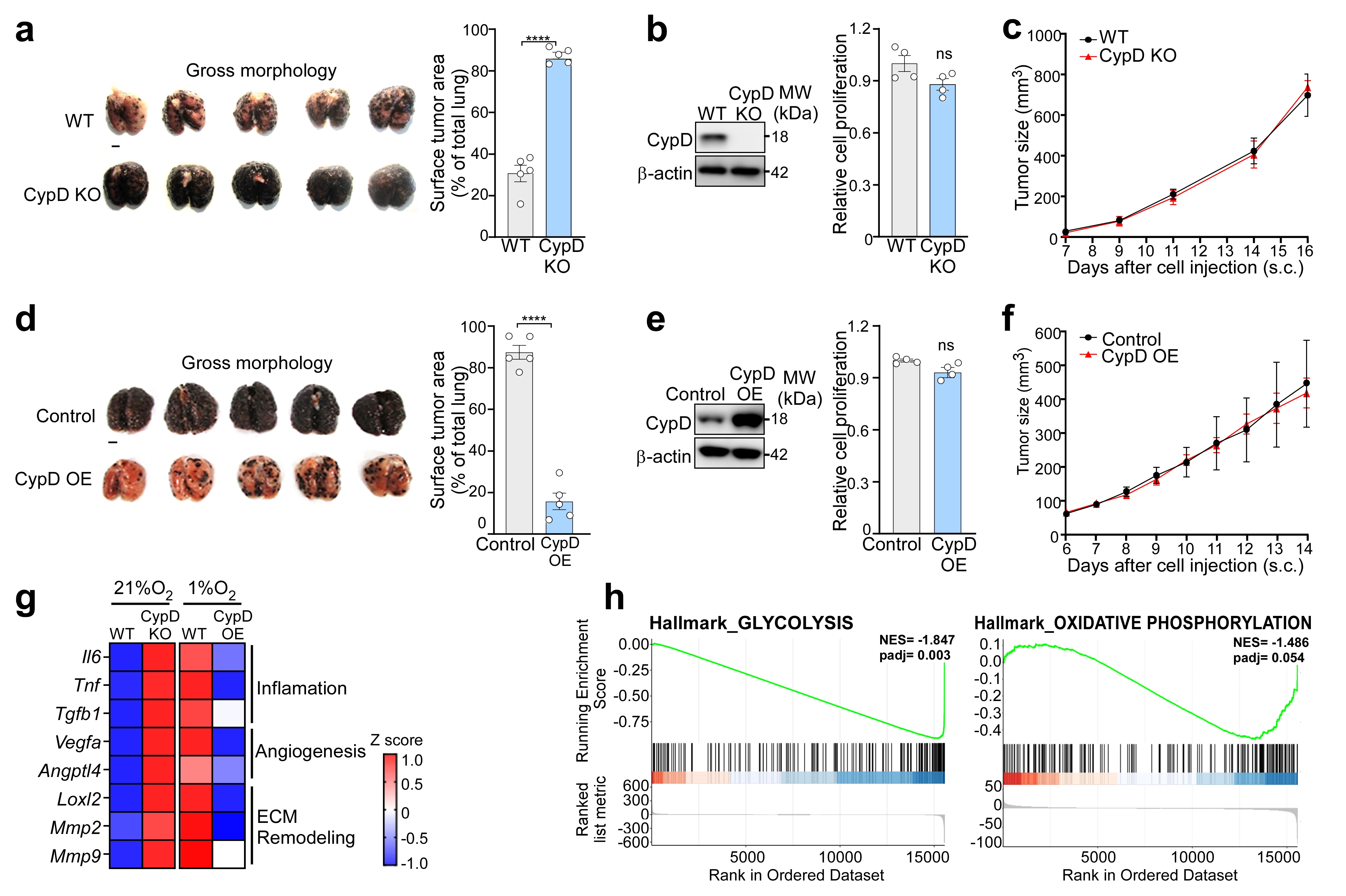


Fig. S1. The effect of CypD expression on lung metastasis and proliferation in melanoma cells.

**a.** Effect of CypD knockout (KO) on lung metastasis. Wild-type (WT) or CypD KO B16F10 cells (1×10^5^ cells) were injected into the tail vein of C57BL/6 mice. After 14 days, the lungs were harvested, fixed, and photographed (left). Metastatic foci on the lung surface were quantified using ImageJ software, and the total area of lung metastasis was expressed in square pixels (right, n=5 per group). Scale bar, 1 mm. **b.** Cell proliferation of CypD KO B16F10 cells. WT and CypD KO B16F10 cells were analyzed by western blotting (left). Serially diluted WT and CypD KO cells were cultured in 96-well plates for 48 hours, and cell proliferation was analyzed using MTT assays and compared (right, n=4). **c**. Tumor growth of CypD KO B16F10 cells. WT and CypD KO B16F10 cells were injected subcutaneously into both flanks of mice, and tumor size was measured using a caliper (n=4 per group). **d**. Effect of CypD overexpression (OE) on lung metastasis. B16F10 cells (5×10^5^ cells) stably transfected with pcDNA (Control) or pcDNA-CypD (CypD OE) plasmids were injected into the tail vein of C57BL/6 mice and analyzed as in (**a**) (n=5 per group). Scale bar, 1 mm. **e**. Proliferation of CypD OE cells. The expression of CypD in Control or CypD OE B16F10 cells was analyzed by western blot (left). Cell proliferation was analyzed as in (**b**), (right, n=4). **f.** Tumor growth of CypD OE B16F10 cells. Control or CypD OE cells were injected subcutaneously into both flanks of mice, and tumor size was measured using a caliper (n=6 per group). **g**. CypD-mediated gene expression changes. RNA sequencing (RNA-seq) was performed to analyze transcriptomic changes in WT and CypD KO cells cultured under 21% oxygen, and in CypD WT and CypD OE cells cultured under 1% oxygen. Z-scores of tumor microenvironment-related genes were visualized as a heatmap. **h**. Gene set enrichment analysis (GSEA)^84^ was performed on RNA-seq data obtained from WT and CypD OE B16F10 cells cultured under 1% oxygen. Major metabolic pathways that were downregulated in CypD OE cells compared to WT cells are presented. Data are presented as the mean ± SEM. ****, *p* < 0.0001; ns, not significant.


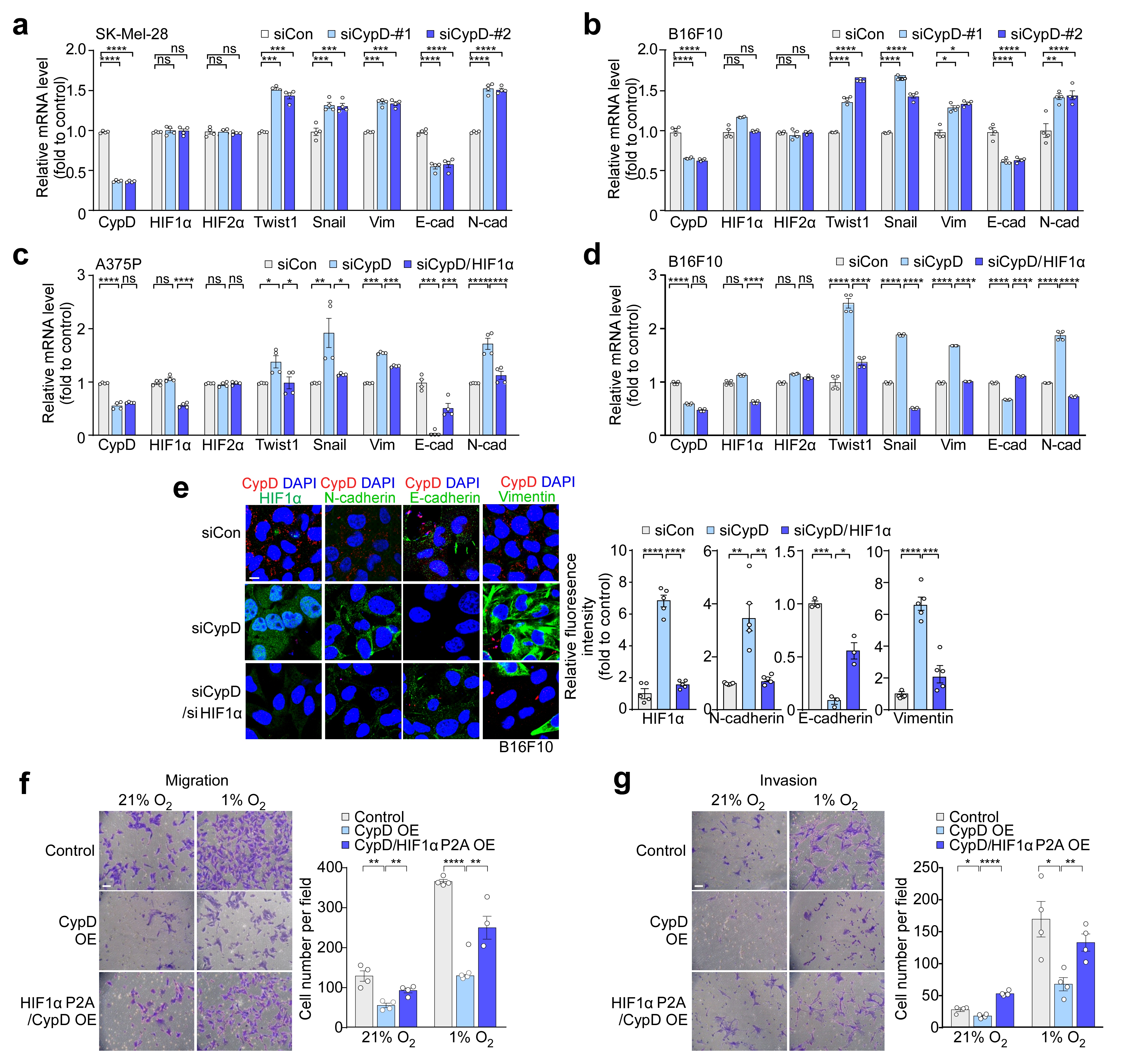


Fig. S2. The effect of CypD on HIF1α-mediated EMT.

**a-b.** Comparison of mRNA levels after CypD siRNA treatment. SK-Mel-28 and B16F10 cells were transfected with control (siCon) or CypD siRNAs (siCypD) for 24 h and analyzed by RT-qPCR (n=4). **c-d.** CypD and HIF1α silencing in A375P and B16F10 cells. Human melanoma A375P and mouse melanoma B16F10 cells were transfected with control (siCon), CypD (siCypD), or HIF1α (siHIF1α) siRNAs as indicated and analyzed by RT-qPCR (n=4). **e**. Immunofluorescence staining for HIF1α and EMT markers. B16F10 cells transfected with control, CypD, or HIF1α siRNAs as indicated were analyzed by immunocytochemistry. Representative immunofluorescence images are shown (left), and the quantification of fluorescence intensity is presented (right, n=5). Scale bar, 10 µm. **f.** Migration upon CypD or HIF1α P2A overexpression. Transwell migration assay was performed using control, CypD or HIF1α P2A overexpressing B16F10 cells under normoxia (21% O_2_) or hypoxia (1% O_2_) for 24 hours. Scale bar, 50 µm. **g.** Invasion upon CypD (CypD OE) or HIF1αP2A (HIF1αP2A OE) overexpression. Transwell invasion assay was performed using control, CypD or HIF1αP2A B16F10 cells under normoxia (21% O_2_) or hypoxia (1% O_2_) for 24 hours. . Scale bar, 50 µm. Data are presented as the mean ± SEM. *, *p* < 0.05; **, *p* < 0.01; ***, *p* < 0.001; ****, *p* < 0.0001; ns, not significant.


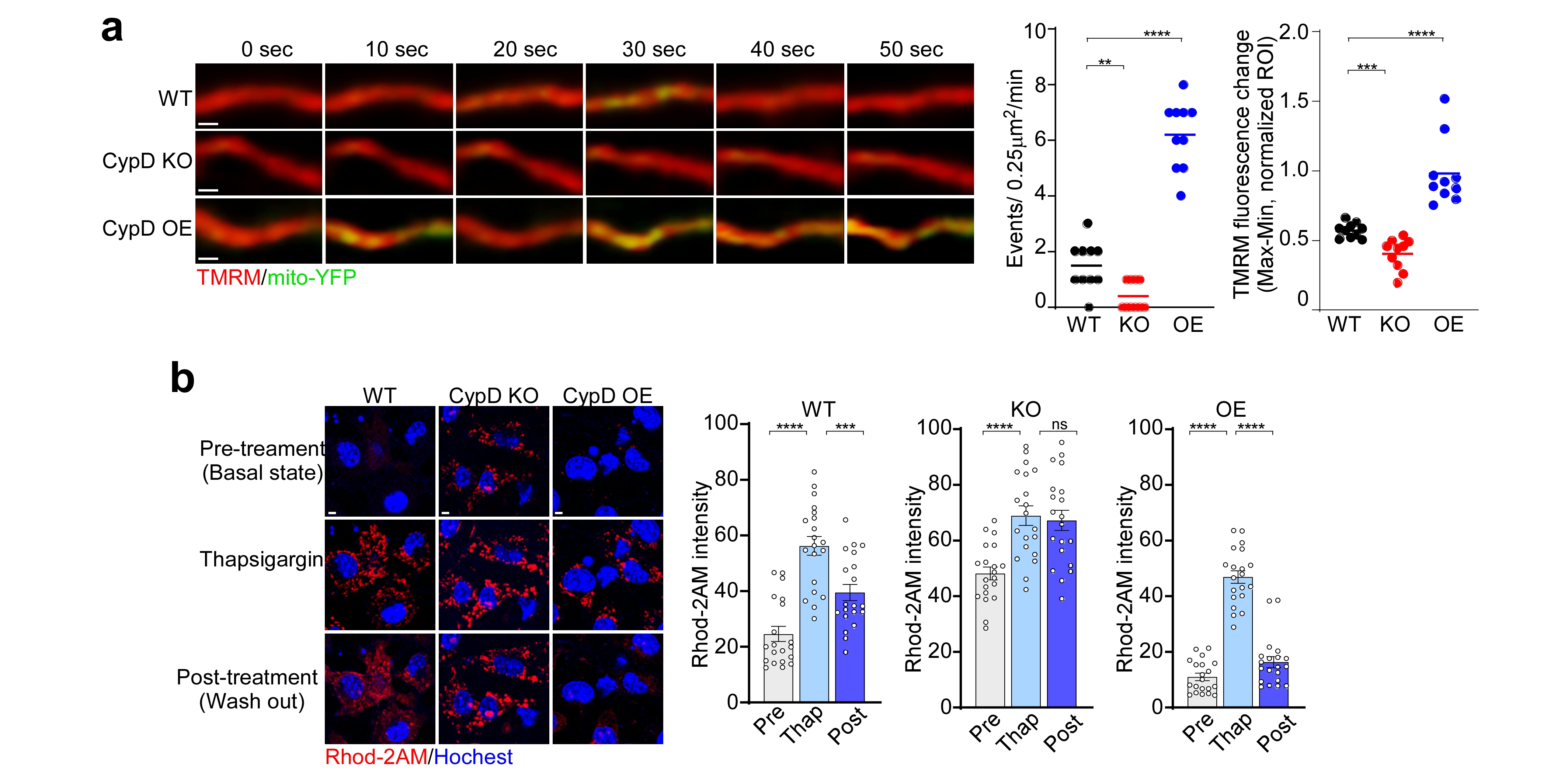


Fig. S3. CypD-mediated mPTP opening and calcium dynamics.

**a**. CypD-mediated mPTP opening. B16F10 WT, CypD KO, and CypD OE cells were transfected with the Mito-YFP construct, stained with TMRM, and analyzed by confocal microscopy (left). Quantification of imaging data was performed using ImageJ (right). Scale bar, 200 nm. **b.** CypD-mediated mitochondrial calcium influx and efflux. B16F10 WT, CypD KO, and CypD OE cells were stained with Rhod-2AM and Hoechst, and subjected to real-time monitoring by confocal microscopy before and after treatment with 10 µM thapsigargin (left). Quantification of imaging data was performed using ImageJ (right). Scale bar, 5 µm. Data are presented as the mean ± SEM. ***, *p* < 0.001; ****, *p* < 0.0001; ns, not significant.


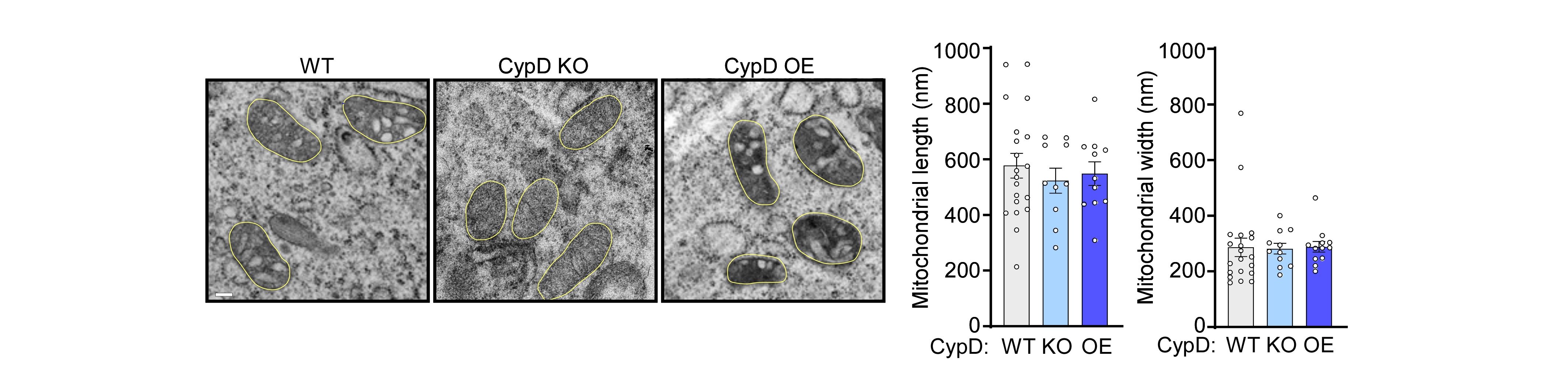


Fig. S4. EM imaging of mitochondria. WT, CypD KO, and CypD OE B16F10 cells were analyzed using Bio-Transmission Electron Microscope (Bio-TEM, left). Mitochondrial length and width were measured and quantified using ImageJ (right). Scale bar, 100 nm.


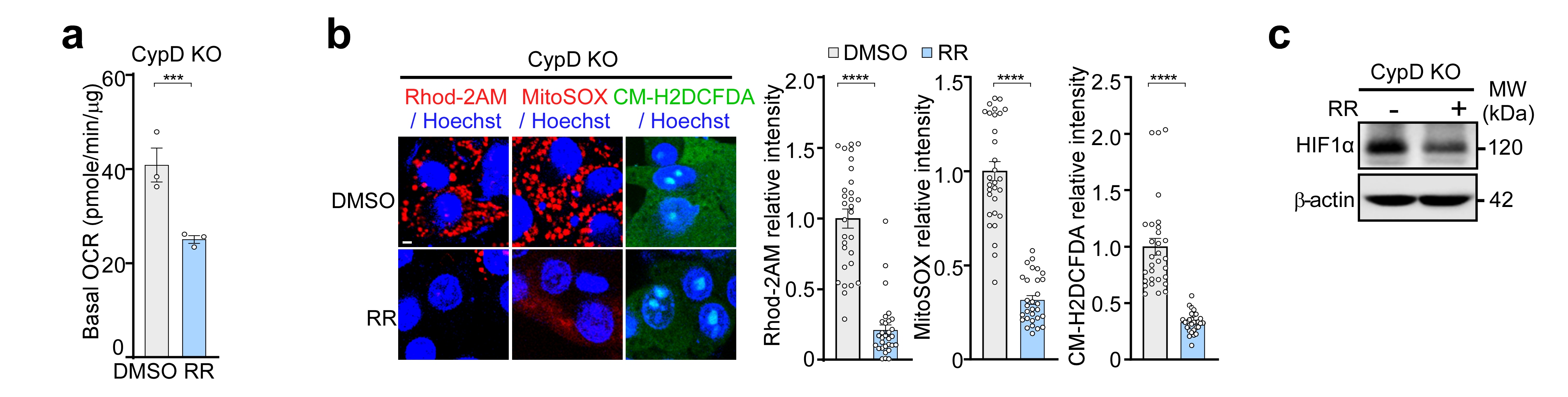


Fig. S5. Effect of mitochondrial calcium levels on mitochondrial respiration, ROS production, and HIF1α stabilization.

**a.** Basal oxygen consumption rate (OCR) following ruthenium red (RR) treatment in CypD KO B16F10 cells. The basal OCR of CypD KO B16F10 cells, treated with DMSO or 10 µM RR for 24 h, was measured using the Seahorse XF analyzer (n=3). **b.** Mitochondrial calcium and ROS levels in CypD KO B16F10 cells treated with ruthenium red (RR). CypD KO B16F10 cells treated with DMSO or 10 µM RR were stained with Rhod-2AM, MitoSOX or CM-H2DCFDA. The cells were analyzed by confocal microscopy. Representative images are shown (left; scale bar, 5 µm), and quantification data are presented (right; n=30). **c**. HIF1α expression following RR treatment in CypD KO cells. CypD KO B16F10 cells were treated with 10µM RR for 24 h and analyzed by western blotting. Data are presented as the mean ± SEM. ***, *p* < 0.001; ****, *p* < 0.0001; ns, not significant.


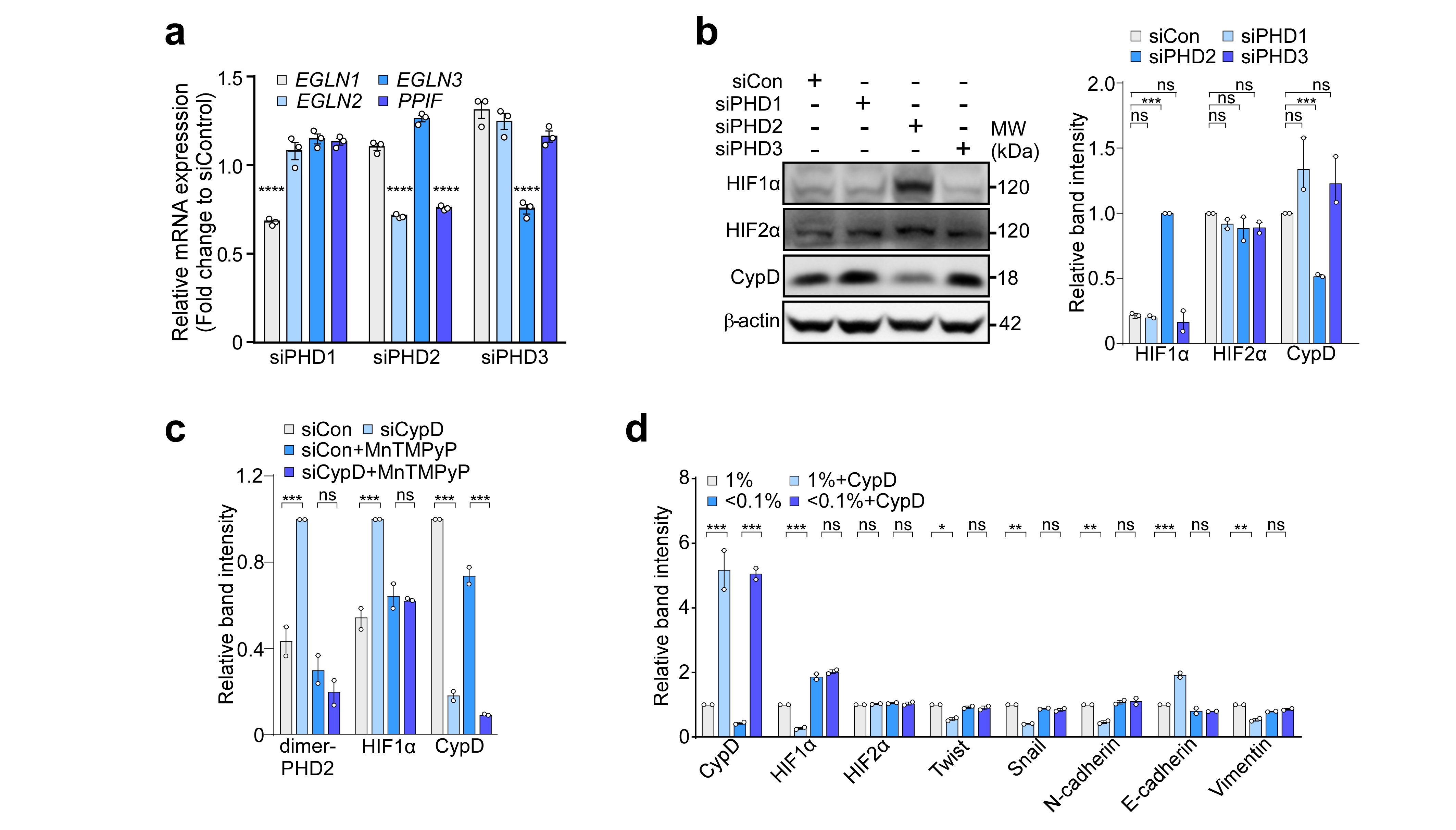


Fig. S6. Effect of HIF prolyl hydroxylase (PHD) isoforms on CypD expression and HIF1α stabilization in melanoma.

**a.** Expression of CypD (*PPIF*) mRNA following PHD isoform-specific siRNA treatment. A375P cells were transfected with control siRNA or PHD1 (*EGLN2*), PHD2 (*EGLN1*), PHD3 (*EGLN3*) siRNAs for 24h. The relative mRNA expression levels compared to control were analyzed by RT-qPCR (n=3). **b.** Western blot analysis following PHD isoform-specific siRNA treatment. A375P cells were transfected with control, PHD1 (*EGLN2*), PHD2 (*EGLN1*), or PHD3 (*EGLN3*) siRNAs and analyzed by western blotting (left). The band intensity was quantified using ImageJ (right). **c.** PHD2 dimerization following CypD silencing and MnTMPyP treatment. Western blotting data from **Figure 4n** were quantified using ImageJ. **d.** Expression of EMT markers and HIF1α after CypD overexpression under mild or severe hypoxia. Western blotting data from **Figure 4o** were quantified using ImageJ. Data are presented as the mean ± SEM. *, *p* < 0.05; **, *p* < 0.01; ***, *p* < 0.001; ****, *p* < 0.0001; ns, not significant.


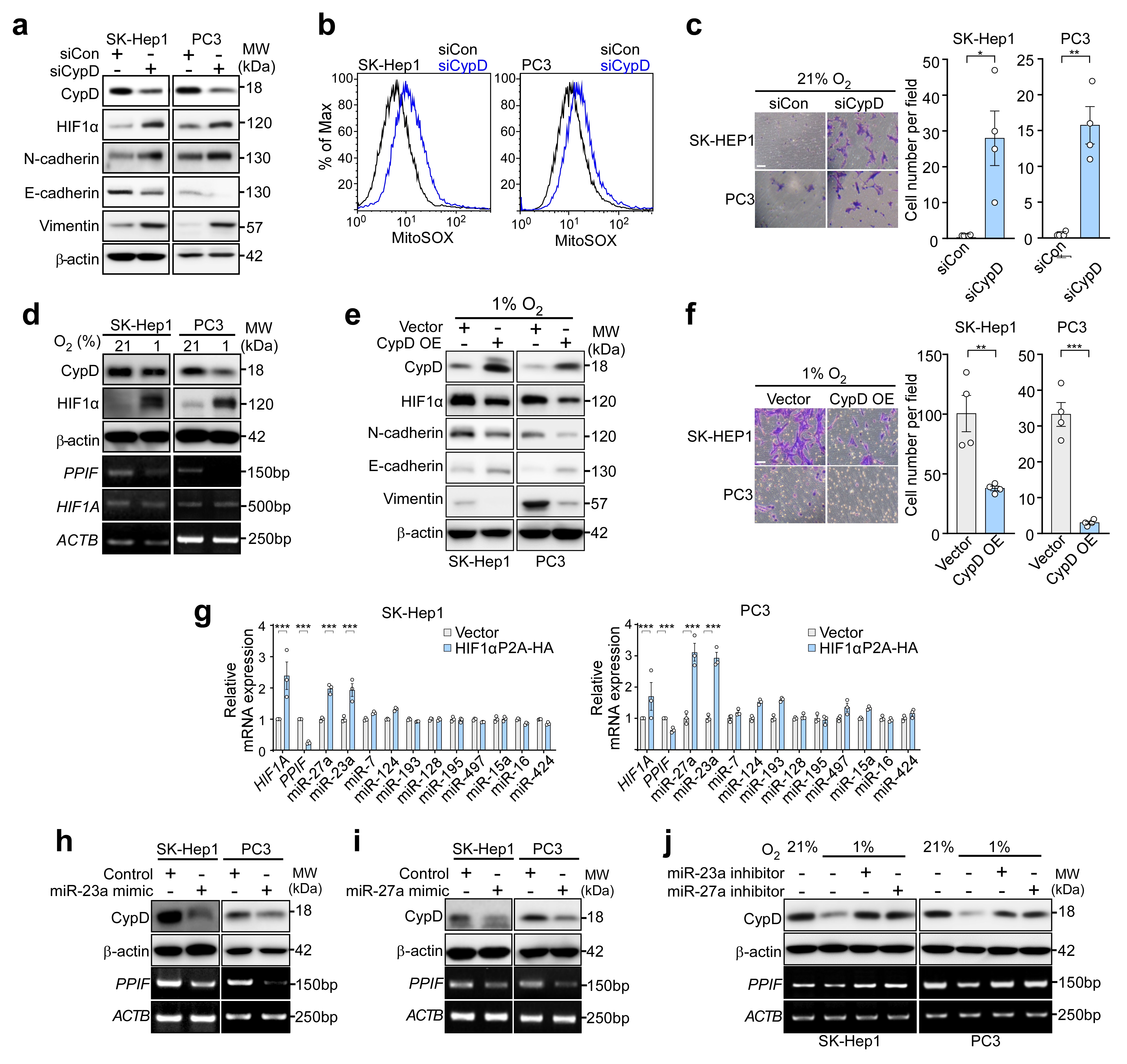


Fig. S7. Conservation of the CypD-HIF1α regulatory mechanism in metastatic liver and prostate cancers

**a.** Expression of HIF1α and EMT markers upon CypD inhibition. SK-Hep1 and PC3 cells were transfected with control or CypD siRNA, harvested, and analyzed by western blotting. **b.** ROS changes upon CypD silencing. SK-Hep1 and PC3 cells transfected with control (siCon) or CypD siRNA(siCypD) were stained with MitoSOX and analyzed by flow cytometry. **c.** Invasion upon CypD silencing. A Transwell invasion assay was performed using SK-Hep1 and PC3 cells transfected with control or CypD siRNA under 21% oxygen for 24 hours. **d.** Expression of HIF1α and CypD under hypoxia. SK-Hep1 and PC3 cells were incubated under 1% or 21% oxygen for 24 hours, harvested, and analyzed by western blotting or RT-PCR. **e.** Expression of HIF1α and EMT markers upon CypD overexpression. SK-Hep1 and PC3 cells transiently transfected with pcDNA (vector) or pcDNA-CypD (CypD) were incubated under 1% oxygen for 24 hours, harvested, and analyzed by western blotting. **f.** Invasion upon CypD overexpression. A Transwell invasion assay was performed using SK-Hep1 and PC3 cells transfected with pcDNA (vector) or pcDNA-CypD (CypD OE) under 1% oxygen for 24 hours. **g.** Expression of miRNAs. SK-Hep1 and PC3 cells were transfected with vehicle or HIF1α P2A, and miRNA expression was analyzed by qPCR. Fold changes were calculated relative to the vehicle control. **h-i.** Effect of miRNA mimics. SK-Hep1 and PC3 cells were transfected with control or miR-23a mimics (**h**), or miR-27a mimics (**i**) for 24 hours and analyzed by western blotting and RT-PCR. **j.** Effect of miRNA inhibitors. SK-Hep1 and PC3 cells were transfected with control or miR-23a and miR-27a inhibitors for 24 hours. CypD protein and RNA expression were analyzed by western blotting and RT-PCR. Data are presented as the mean ± SEM. *, *p* < 0.05; **, *p* < 0.01; ***, *p* < 0.001.


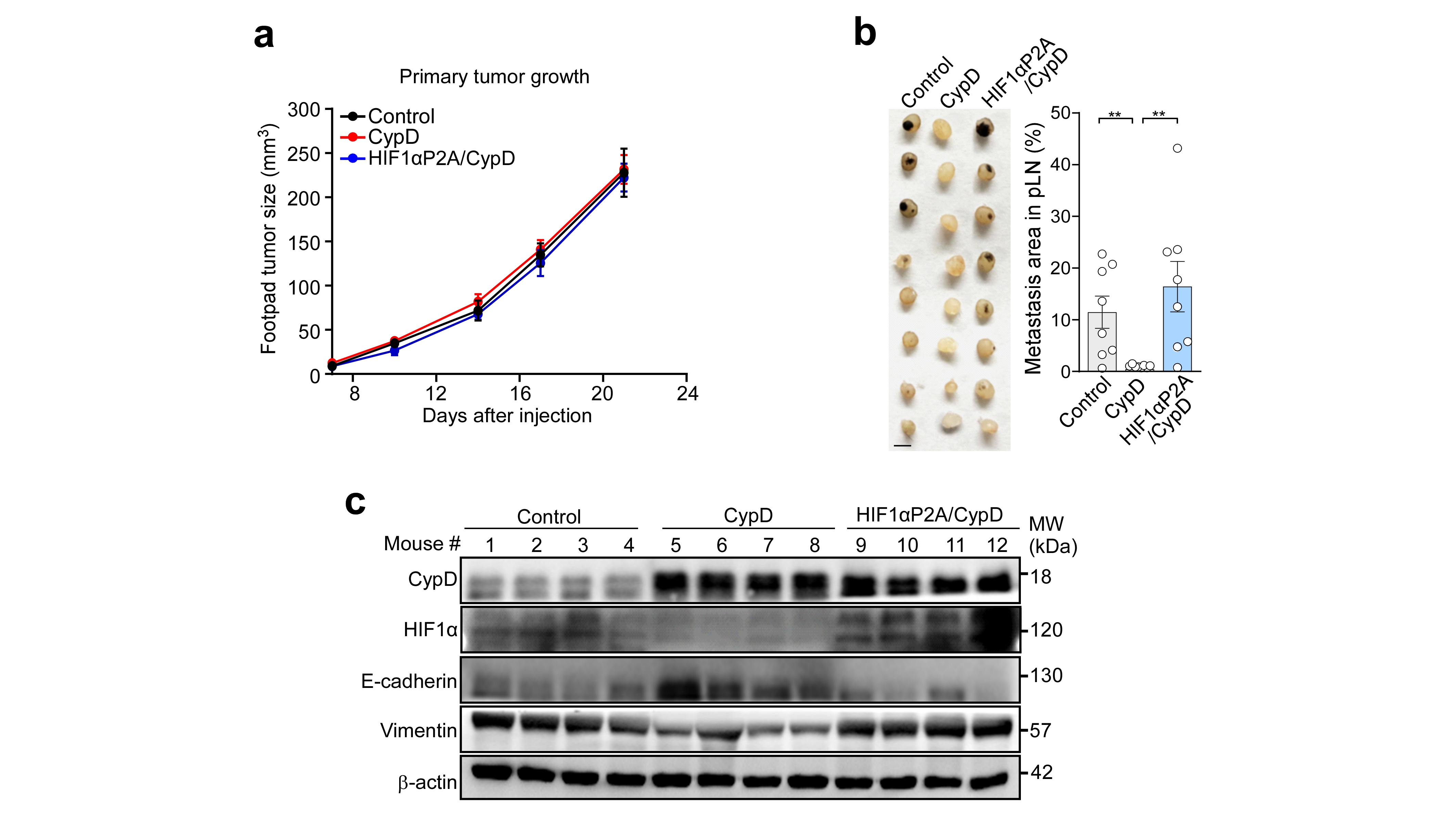


Fig. S8. Metastatic suppression by CypD OE can be reversed by HIF1α

**a.** Primary tumor growth. B16F10 cells overexpressing CypD alone (CypD) or co-expressing CypD and HIF1α P2A (CypD/HIF1α P2A) were injected subcutaneously into the footpads of mice, and tumor growth was measured by caliper. **b.** Metastasis to lymph nodes. Popliteal lymph nodes were isolated, and their gross appearances were compared and quantified. Scale bar, 1 mm. **c.** Western blot analysis. Primary tumor tissues isolated from the B16F10 tumors were analyzed by western blotting. Data are presented as the mean ± SEM. **, *p* < 0.01.


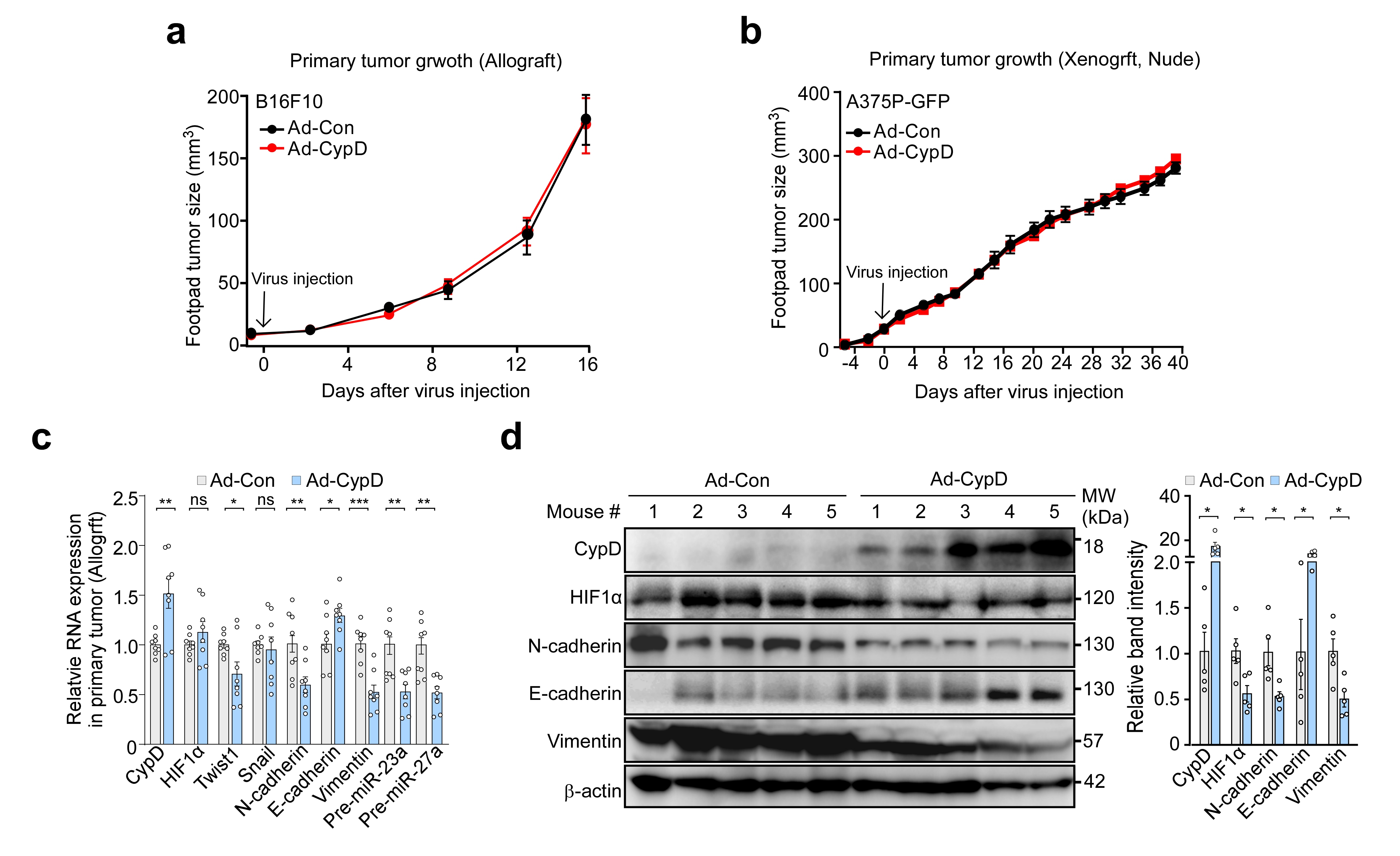


Fig. S9. Antimetastatic activity of intratumoral injection of Ad-CypD

**a-b**. Tumor growth following intratumoral injection of adenovirus expressing CypD (Ad-CypD). B16F10 (**a**) and A375P-GFP (**b**) cells were injected subcutaneously into the footpads of C57BL/6 and nude mice, respectively. After one week, either a control adenovirus (Ad-Con) or a CypD-expressing adenovirus (Ad-CypD) was administered intratumorally. Tumor growth was measured using calipers (n = 10 tumors, 5 mice per group). **c-d**. RNA and protein expression. Tissues isolated from B16F10 tumors were analyzed by RT-qPCR (**c**; n = 5) and western blotting (**d**; n = 5). Data are presented as the mean ± SEM. *, *p* < 0.05; **, *p* < 0.01; ***, *p* < 0.005; ns, not significant.


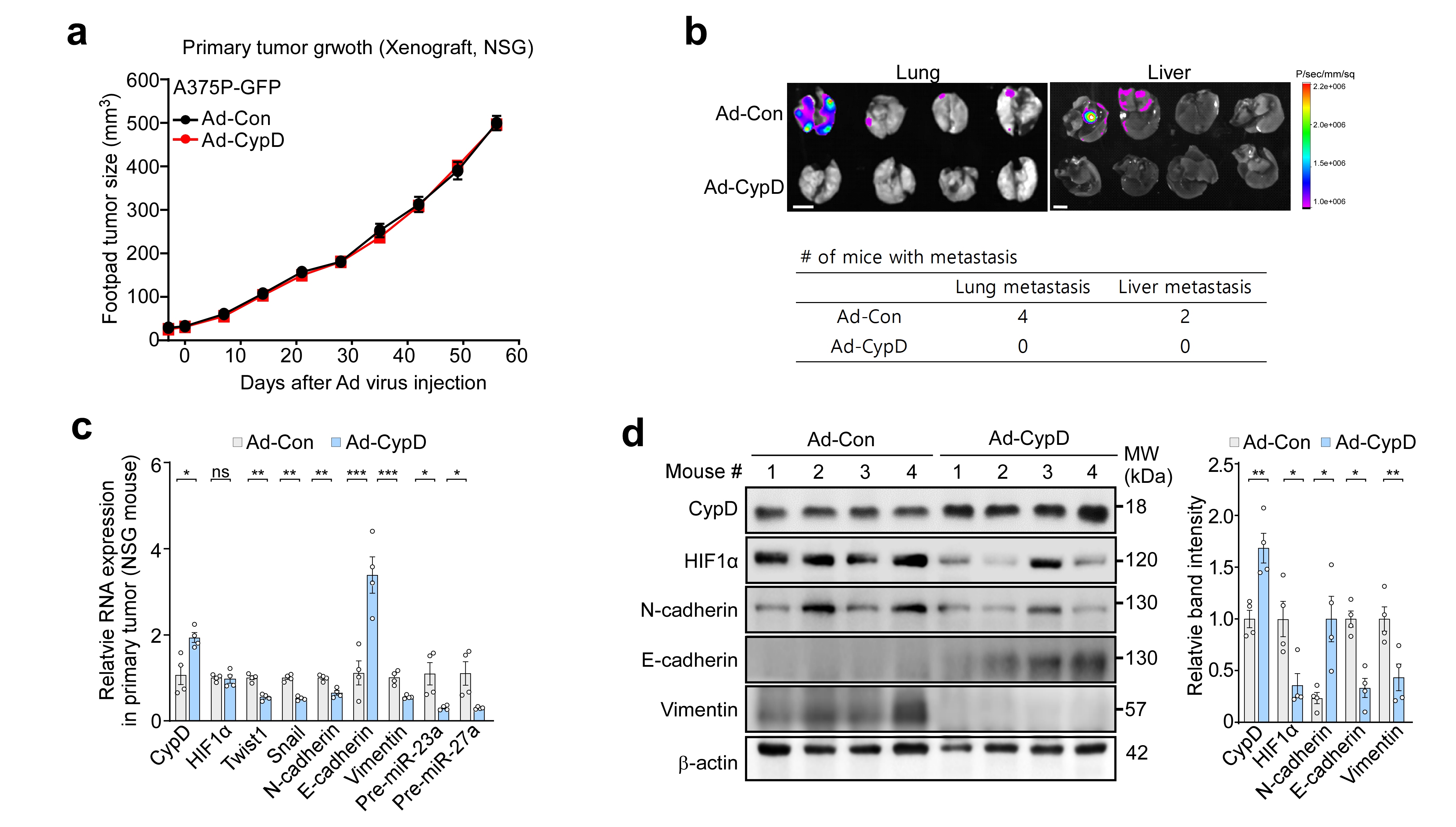


Fig. S10. Suppression of lung and liver metastasis by intratumoral injection of Ad-CypD

**a.** Primary tumor growth. A375P-GFP cells were injected subcutaneously into the footpads of NSG mice (n = 8 tumors, 4 mice per group), and tumor volume was measured using a caliper. After one week, either a control adenovirus (Ad-Con) or a CypD-overexpressing adenovirus (Ad-CypD) was administered intratumorally. **b.** Metastasis to the lung and liver. At the end of the experiment in (**a**), the lung and liver were isolated and analyzed using the In Vivo Xtreme bioimaging system to detect GFP signals (n = 4 per group). Scale bar, 1 cm. **c–d.** RNA and protein expression. Tumor tissues isolated from the xenograft model in (**a**) were analyzed by RT-qPCR (**c**; n = 4) and western blotting (**d**; n = 4). Data are presented as the mean ± SEM. *, *p* < 0.05; **, *p* < 0.01; ***, *p* < 0.005; ns, not significant.


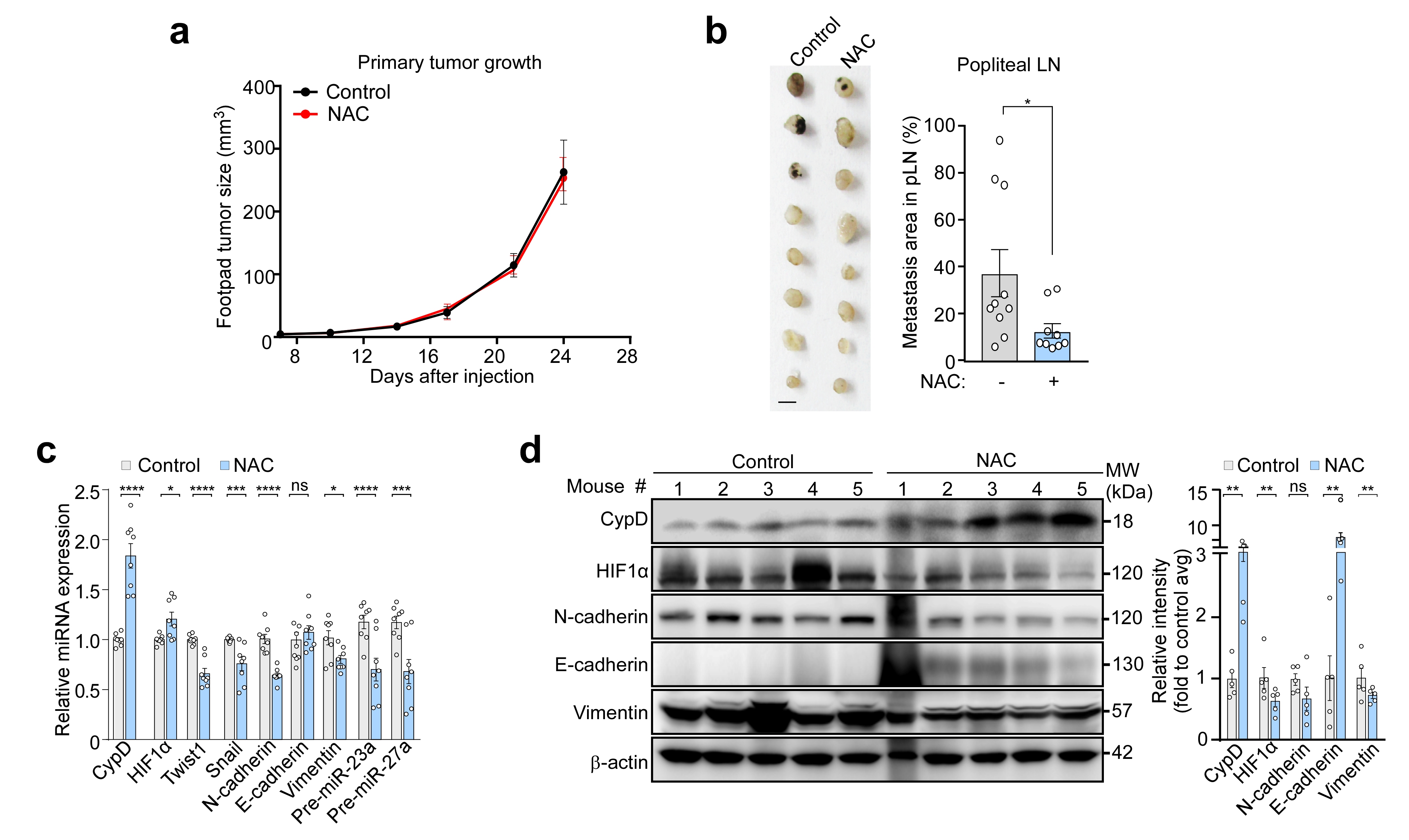


Fig. S11. Anti-metastatic activity of oral administration of the antioxidant NAC

**a.** Primary tumor growth. B16F10 cells (2 × 10⁵) were injected subcutaneously into the footpads of mice, and the mice received drinking water supplemented with NAC (1 g/L) or plain water (Control) for 24 days. Tumor growth was measured using a caliper. **b.** Metastasis to the lymph nodes. After 24 days of cancer cell injection, mice were sacrificed, and the popliteal lymph nodes (pLNs) and footpads containing tumors were isolated. Metastatic areas were assessed based on the extent of pigmented regions. Scale bar, 1 mm. **c-d.** RNA and protein expression. Primary tumor tissues were isolated and analyzed by RT-qPCR (**c**) and western blotting (**d**). Data are presented as the mean ± SEM. *, *p* < 0.05; **, *p* < 0.01; ***, *p* < 0.005; ****, *p* < 0.001; ns, not significant.


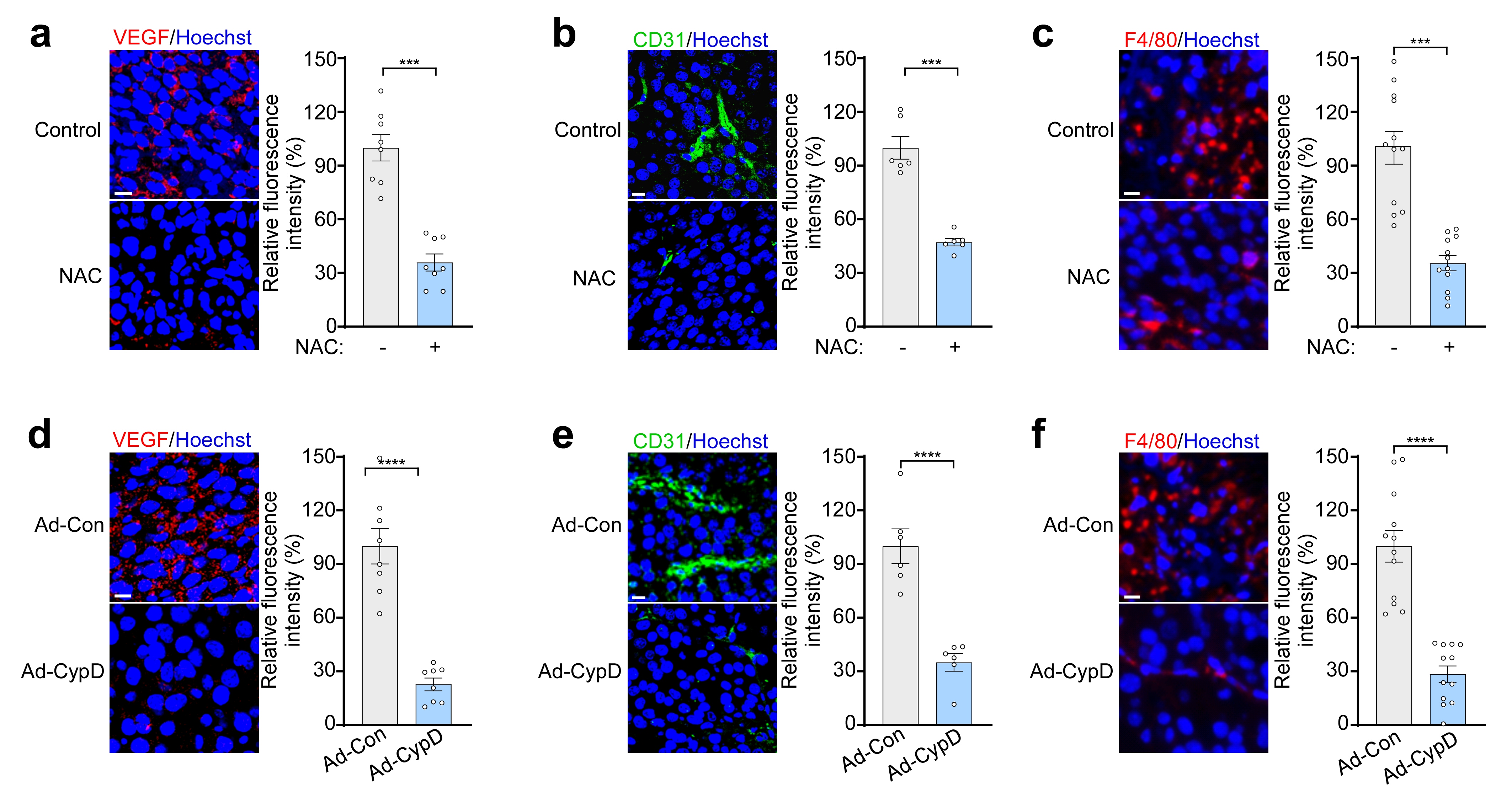


Fig. S12. Microenvironmental change of oral administration of the antioxidant NAC or intratumoral injection of Ad-CypD

**a-c.** Effects of NAC on the tumor tissue microenvironment. B16F10 cells (2×10⁵) were injected subcutaneously into the footpads of mice, which were then given drinking water supplemented with NAC (1 g/L) or plain water (Control) for 24 days. Tumors were harvested, fixed, paraffin-embedded, and sectioned, followed by immunofluorescence staining with antibodies against VEGF (**a**), CD31 (**b**), and F4/80 (**c**). Representative images (left) and corresponding quantification (right) are shown. **d-f.** Effects of CypD OE on the tumor tissue microenvironment. B16F10 cells (2×10⁵) were injected subcutaneously into the footpads of mice, followed by intratumoral injection of Ad-Con or Ad-CypD. Tumors were analyzed as above. Data are presented as the mean ± SEM. ***, *p* < 0.005; ****, *p* < 0.001.

Table S1. Primer sequences for reverse transcription-quantitative PCR (RT-qPCR) and mutated sequences.

| Gene | Accession  number | Species | Forward | Reverse |
| --- | --- | --- | --- | --- |
| *PPIF* | *NM_005729.4* | *Human* | 5’-GCTCGTGTACCTGGACGTG | 5’-AGCTCAACTGGCCACAGTCT |
| *HIF1A* | *NM_001530.4* | *Human* | 5’-TTCCAGTTACGTTCCTTCGATCA | 5’-TTTGAGGACTTGCGCTTTCA |
| *EPAS1*  *(HIF2A)* | *NM_001430.5* | *Human* | 5’-AAGCCTTGGAGGGTTTCATT | 5’-TGCTGGATTGGTTCACACAT |
| *TWIST1* | *NM_000474.4* | *Human* | 5’-GCCAGGTACATCGACTTCCTCT | 5’-TCCATCCTCCAGACCGAGAAGG |
| *SNAI1* | *NM_005985.4* | *Human* | 5’-TGCCCTCAAGATGCACATCCGA | 5’-GGGACAGGAGAAGGGCTTCTC |
| *VIM* | *NM_003380.5* | *Human* | 5’-AGGCAAAGCAGGAGTCCACTGA | 5’-ATCTGGCGTTCCAGGGACTCAT |
| *CDH1* | *NM_004360.5* | *Human* | 5’-GCCTCCTGAAAAGAGAGTGGAAG | 5’-TGGCAGTGTCTCTCCAAATCCG |
| *CDH2* | *NM_001792.5* | *Human* | 5’- CCTCCAGAGTTTACTGCCATGAC | 5’-GTAGGATCTCCGCCACTGATTC |
| *ACTB* | *NM_001101.5* | *Human* | 5’-CATGTACGTTGCTATCCAGGC | 5’-CTCCTTAATGTCACGCACGAT |
| *Precursor miR 23a* | *NR_036515.2* | *Human* | 5’-GTTCCTGGGGATGGGATT | 5’-GTTGGAAATCCCTGGCAAT |
| *Precursor miR 27a* | *NR_036515.2* | *Human* | 5’-GAGGAGCAGGGCTTAGCTG | 5’-CGGAACTTAGCCACTGTGAA |
| *Ppif* | *NM_134084.1* | *Mouse* | 5’- GCAGATGTCGTGCCAAAGACTG | 5’- GCCATTGTGGTTGGTGAAGTCG |
| *Hif1a* | *NM_001313919.2* | *Mouse* | 5’-TGCTCATCAGTTGCCACTTC | 5’- CTTCCACGTTGCTGACTTGA |
| *Epas1* | *NM_010137.3* | *Mouse* | 5’-GGACAGCAAGACTTTCCTGAGC | 5’-GGTAGAACTCATAGGCAGAGCG |
| *Twist1* | *NM_011658.2* | *Mouse* | 5’-GATTCAGACCCTCAAACTGGCG | 5’-AGACGGAGAAGGCGTAGCTGAG |
| *Snai1* | *NM_011427.3* | *Mouse* | 5’-TGTCTGCACGACCTGTGGAAAG | 5’- CTTCACATCCGAGTGGGTTTGG |
| *Vim* | *NM_011701.4* | *Mouse* | 5’-CGGAAAGTGGAATCCTTGCAGG | 5’-AGCAGTGAGGTCAGGCTTGGAA |
| *Cdh1* | *NM_009864.3* | *Mouse* | 5’-GGTCATCAGTGTGCTCACCTCT | 5’-GCTGTTGTGCTCAAGCCTTCAC |
| *Cdh2* | *NM_007664.5* | *Mouse* | 5’-CCTCCAGAGTTTACTGCCATGAC | 5’-CCACCACTGATTCTGTATGCCG |
| *Actb* | *NM_007393.5* | *Mouse* | 5’-GTGGGAATGGGTCAGAAGGA | 5’-GTCTCCGGAGTCCATCACAA |
| *Precursor miR-23a* | *NR_029740.1* | *Mouse* | 5’-GTTCCTGGGGATGGGATT | 5’-AGTTGGAAATCCCTGGCAAT |
| *Precursor miR-27a* | *NR_029746.1* | *Mouse* | 5’-CCTGAGGAGCAGGGCTTAG | 5’-CGGAACTTAGCCACTGTGAA |

Table S2. Hallmark pathways enriched in CypD KO compared with WT cells.

The data represents a list of significantly upregulated gene sets from gene set enrichment analysis (GSEA) in CypD KO compared with WT B16F10 cells. The table includes the size of each gene set (setSize), the normalized enrichment score (NES), p-values (pval), p-values adjusted using the Benjamini-Hochberg procedure (p.adj), and q-values (qval).

| Description | setSize | Enrichment  Score | NES | pval | p.adj | qval |
| --- | --- | --- | --- | --- | --- | --- |
| HALLMARK_HYPOXIA | 181 | 0.794635 | 2.727141 | 0.0001 | 0.0005 | 0.0002 |
| HALLMARK_EPITHELIAL_MESENCHYMAL  _TRANSITION | 177 | 0.718068 | 2.454448 | 0.0001 | 0.0005 | 0.0002 |
| HALLMARK_TNFA_SIGNALING_VIA_NFKB | 183 | 0.681466 | 2.339865 | 0.0001 | 0.0005 | 0.0002 |
| HALLMARK_COAGULATION | 103 | 0.704859 | 2.255634 | 0.0001 | 0.0005 | 0.0002 |
| HALLMARK_INTERFERON_GAMMA_RESPONSE | 167 | 0.620435 | 2.111433 | 0.0001 | 0.0005 | 0.0002 |
| HALLMARK_INTERFERON_ALPHA_RESPONSE | 92 | 0.669336 | 2.099091 | 0.0001 | 0.0005 | 0.0002 |
| HALLMARK_APOPTOSIS | 147 | 0.622145 | 2.085995 | 0.0001 | 0.0005 | 0.0002 |
| HALLMARK_IL2_STAT5_SIGNALING | 173 | 0.607191 | 2.073003 | 0.0001 | 0.0005 | 0.0002 |
| HALLMARK_ANGIOGENESIS | 25 | 0.825347 | 2.048202 | 0.0001 | 0.0005 | 0.0002 |
| HALLMARK_P53_PATHWAY | 186 | 0.592831 | 2.041058 | 0.0001 | 0.0005 | 0.0002 |
| HALLMARK_MYOGENESIS | 177 | 0.583474 | 1.994386 | 0.0001 | 0.0005 | 0.0002 |
| HALLMARK_COMPLEMENT | 163 | 0.573803 | 1.946591 | 0.0001 | 0.0005 | 0.0002 |
| HALLMARK_GLYCOLYSIS | 175 | 0.562901 | 1.922517 | 0.0001 | 0.0005 | 0.0002 |
| HALLMARK_KRAS_SIGNALING_UP | 165 | 0.538598 | 1.830167 | 0.0001 | 0.0005 | 0.0002 |
| HALLMARK_UV_RESPONSE_DN | 140 | 0.544859 | 1.817681 | 0.0001 | 0.0005 | 0.0002 |
| HALLMARK_APICAL_JUNCTION | 172 | 0.529153 | 1.804608 | 0.0001 | 0.0005 | 0.0002 |
| HALLMARK_INFLAMMATORY_RESPONSE | 150 | 0.521143 | 1.752515 | 0.0005 | 0.0012 | 0.0004 |
| HALLMARK_TGF_BETA_SIGNALING | 52 | 0.60369 | 1.723634 | 0.0032 | 0.0061 | 0.0023 |
| HALLMARK_IL6_JAK_STAT3_SIGNALING | 68 | 0.574938 | 1.71318 | 0.0023 | 0.0044 | 0.0016 |
| HALLMARK_HEME_METABOLISM | 180 | 0.497549 | 1.70566 | 0.0003 | 0.0008 | 0.0003 |
| HALLMARK_ESTROGEN_RESPONSE_EARLY | 172 | 0.458806 | 1.564699 | 0.0016 | 0.0033 | 0.0012 |
| HALLMARK_PROTEIN_SECRETION | 92 | 0.476813 | 1.495322 | 0.0119 | 0.0212 | 0.0080 |
| HALLMARK_CHOLESTEROL_HOMEOSTASIS | 71 | 0.492546 | 1.478655 | 0.0229 | 0.0370 | 0.0140 |
| HALLMARK_ANDROGEN_RESPONSE | 93 | 0.448617 | 1.409568 | 0.0292 | 0.0457 | 0.0173 |
| HALLMARK_ESTROGEN_RESPONSE_LATE | 167 | 0.396261 | 1.348536 | 0.0224 | 0.0370 | 0.0140 |
| HALLMARK_MITOTIC_SPINDLE | 199 | 0.362443 | 1.258066 | 0.0519 | 0.0787 | 0.0298 |
| HALLMARK_UNFOLDED_PROTEIN_RESPONSE | 107 | -0.43271 | -1.42374 | 0.0145 | 0.0250 | 0.0095 |
| HALLMARK_MTORC1_SIGNALING | 194 | -0.42495 | -1.50644 | 0.0008 | 0.0018 | 0.0006 |
| HALLMARK_DNA_REPAIR | 147 | -0.46878 | -1.61021 | 0.0006 | 0.0014 | 0.0005 |
| HALLMARK_MYC_TARGETS_V2 | 57 | -0.73675 | -2.20781 | 0.0002 | 0.0005 | 0.0002 |
| HALLMARK_G2M_CHECKPOINT | 197 | -0.65336 | -2.31867 | 0.0002 | 0.0005 | 0.0002 |
| HALLMARK_E2F_TARGETS | 198 | -0.7144 | -2.53801 | 0.0002 | 0.0005 | 0.0002 |
| HALLMARK_MYC_TARGETS_V1 | 195 | -0.79321 | -2.81388 | 0.0002 | 0.0005 | 0.0002 |
