# Supplementary material for: Pseudohypoxic stabilization of HIF1α via cyclophilin D suppression promotes melanoma metastasis
Source: Signal Transduct Target Ther. 2025 Jul 24;10:231. doi: 10.1038/s41392-025-02314-8 (PMC12287346; doi:10.1038/s41392-025-02314-8)

Supplementary Materials for

Pseudohypoxic stabilization of HIF1α via cyclophilin D suppression promotes melanoma metastasis

**Authors:** Hye-Kyung Park^1^*, Sung Hu^1^, So Yeon Kim^1^, Sora Yoon^2^, Nam Gu Yoon^1^, Ji Hye Lee^1^, Wonyoung Choi^3,4^, Sun-Young Kong^3,5^, Jong Heon Kim^3,6^, Dougu Nam^1^, and Byoung Heon Kang^1^*

Correspondence to: Hye-Kyung Park ([hkparkgene@unist.ac.kr](mailto:hkparkgene@unist.ac.kr)) and Byoung Heon Kang ([kangbh@unist.ac.kr](mailto:kangbh@unist.ac.kr))

**This file includes:**

Original immunoblot films

Original immunoblot films


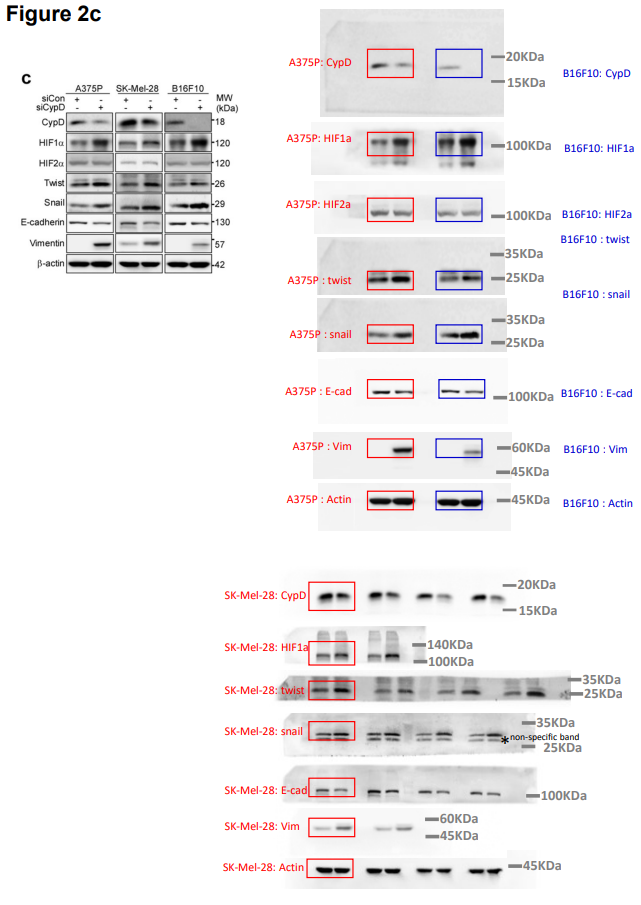


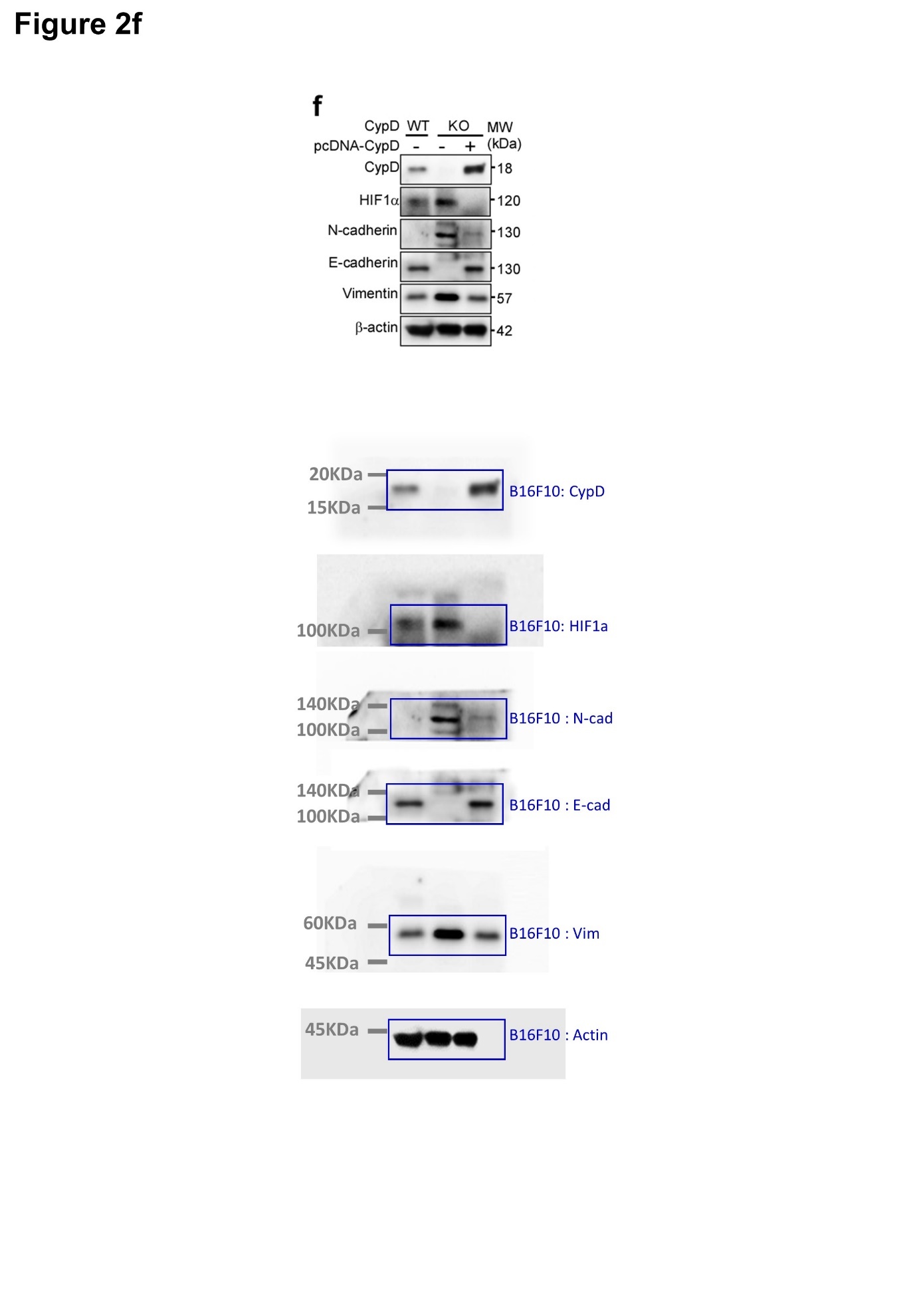


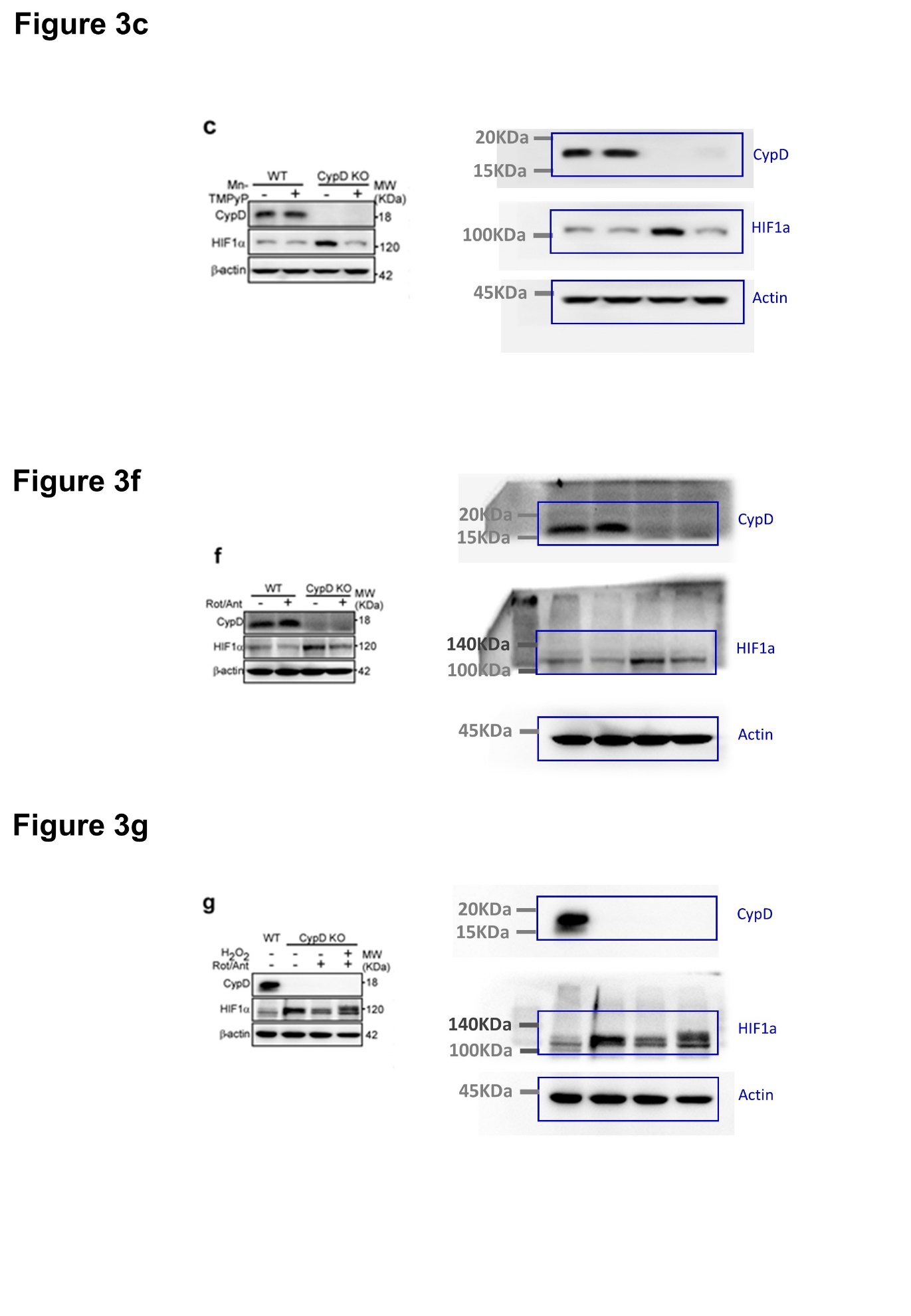


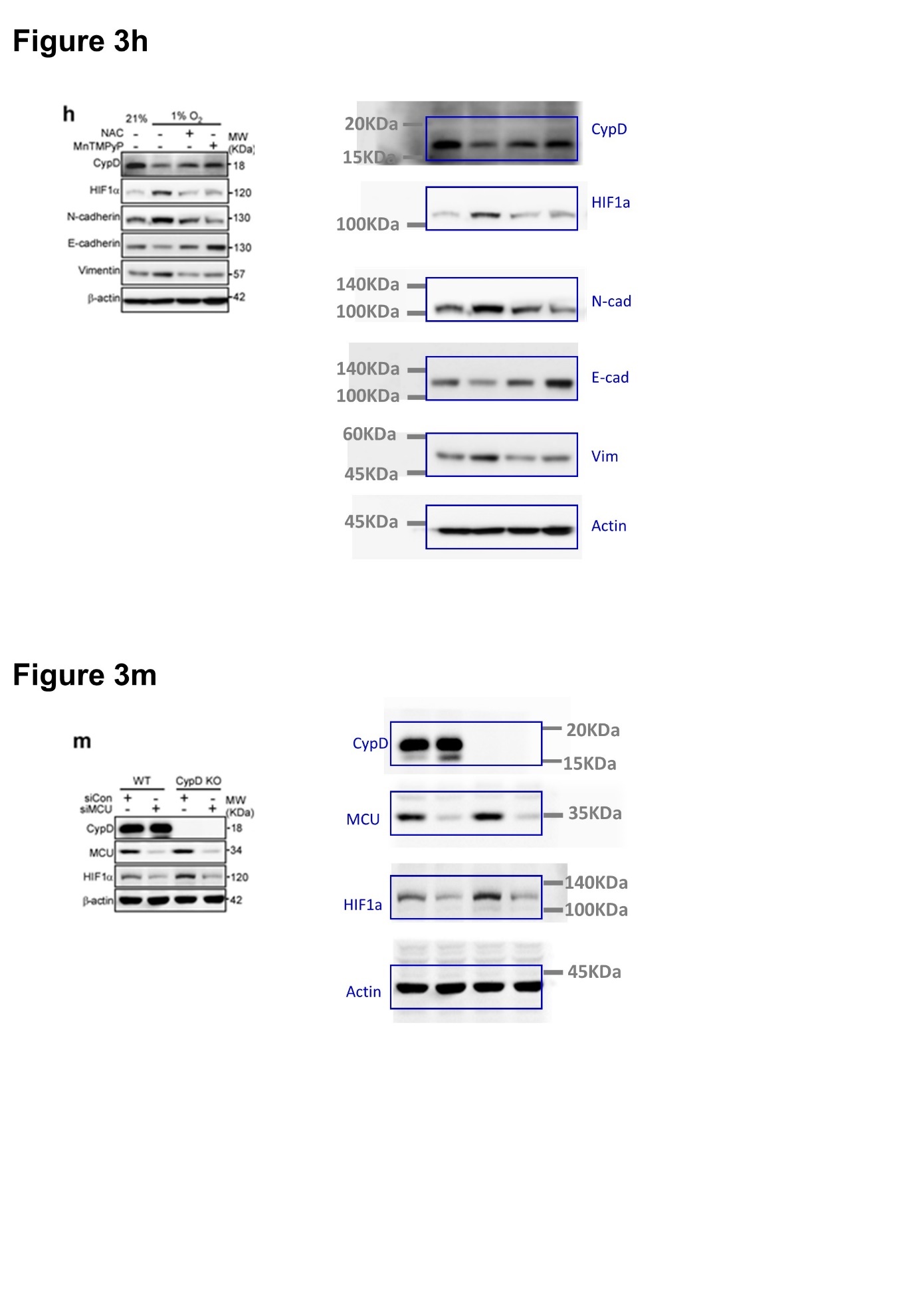


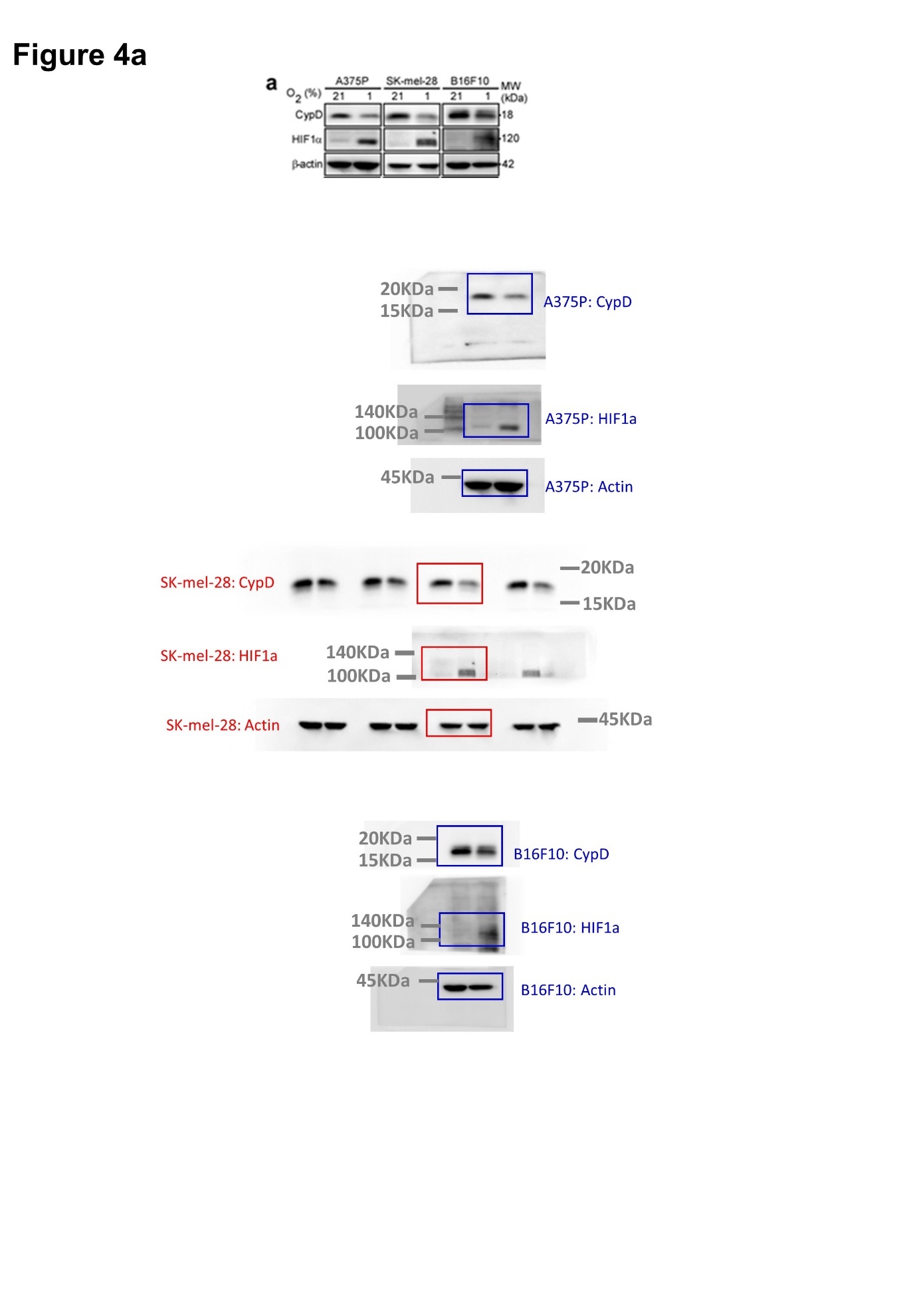


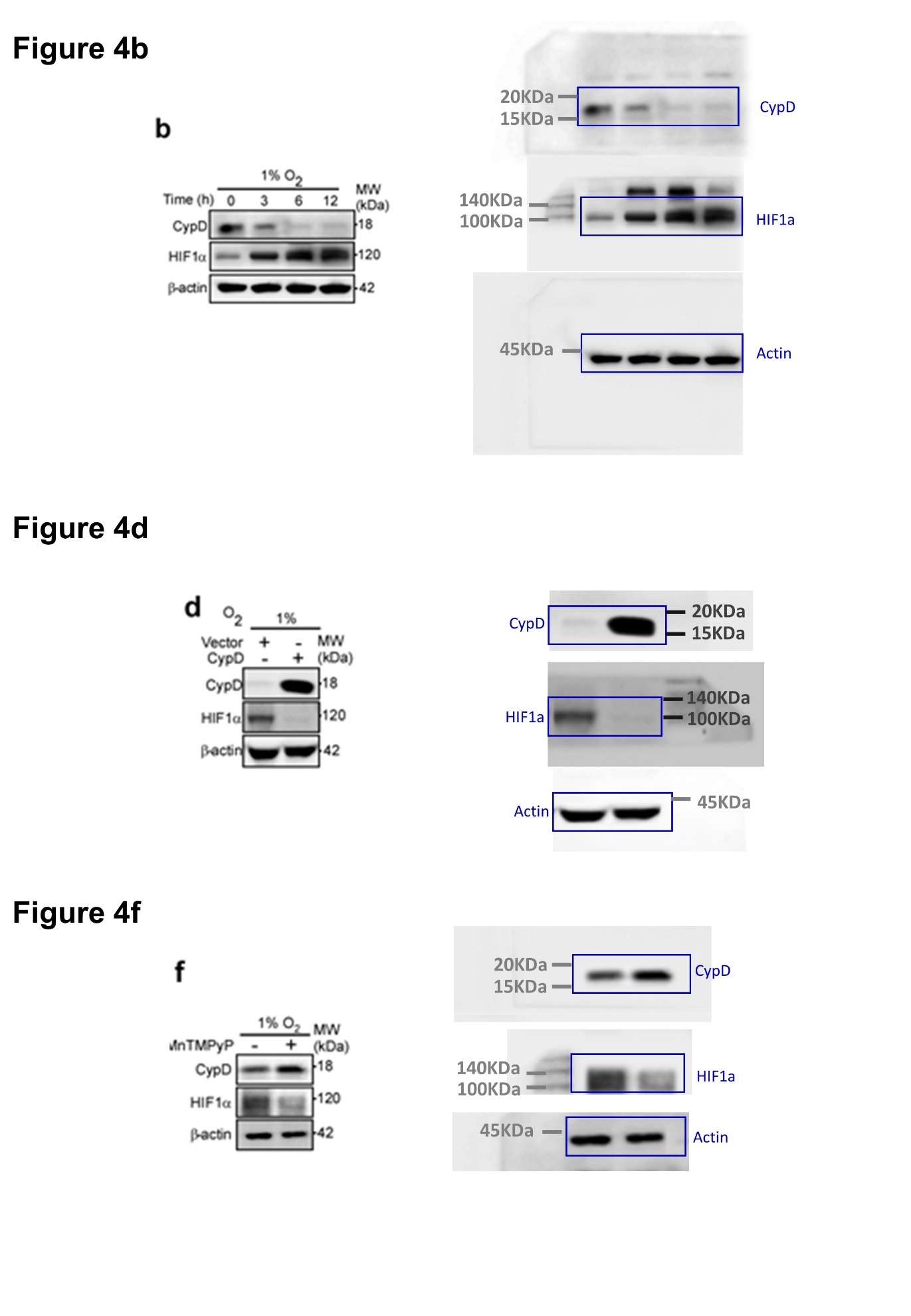


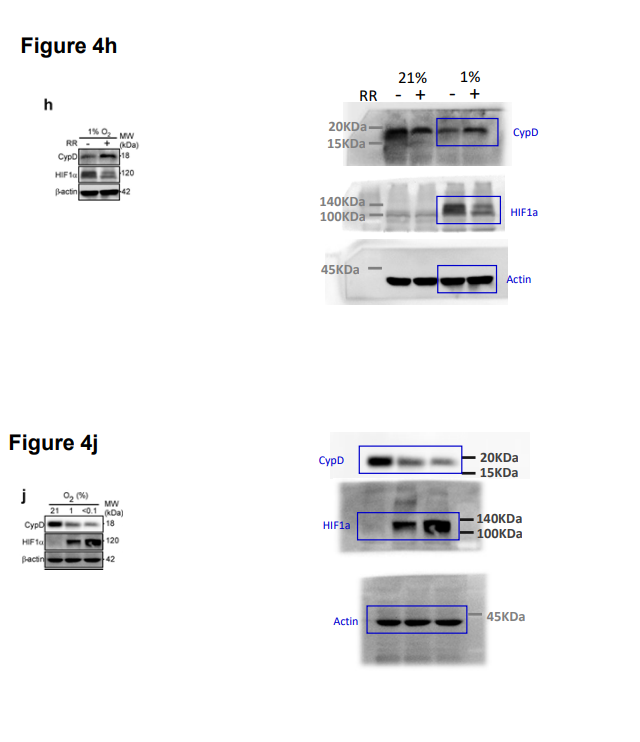


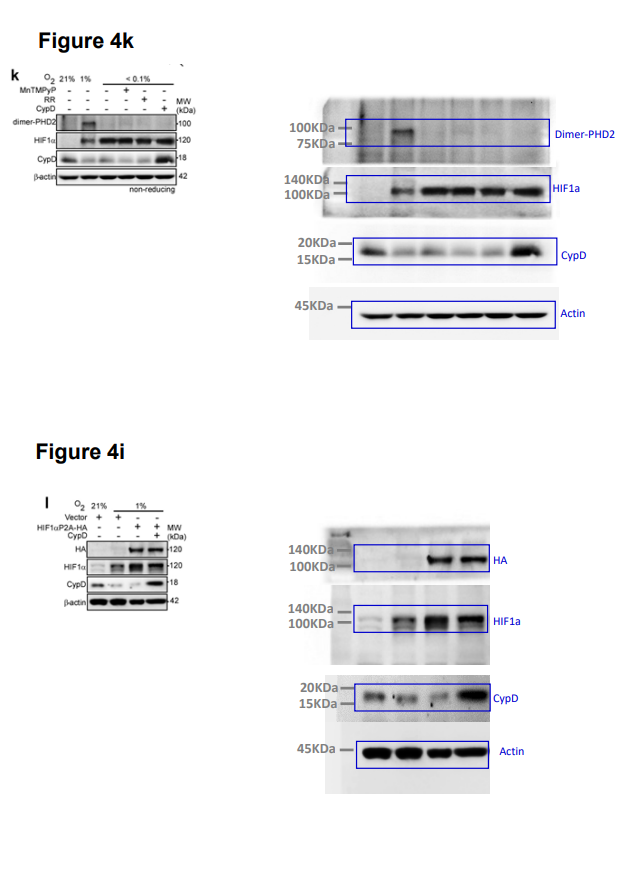


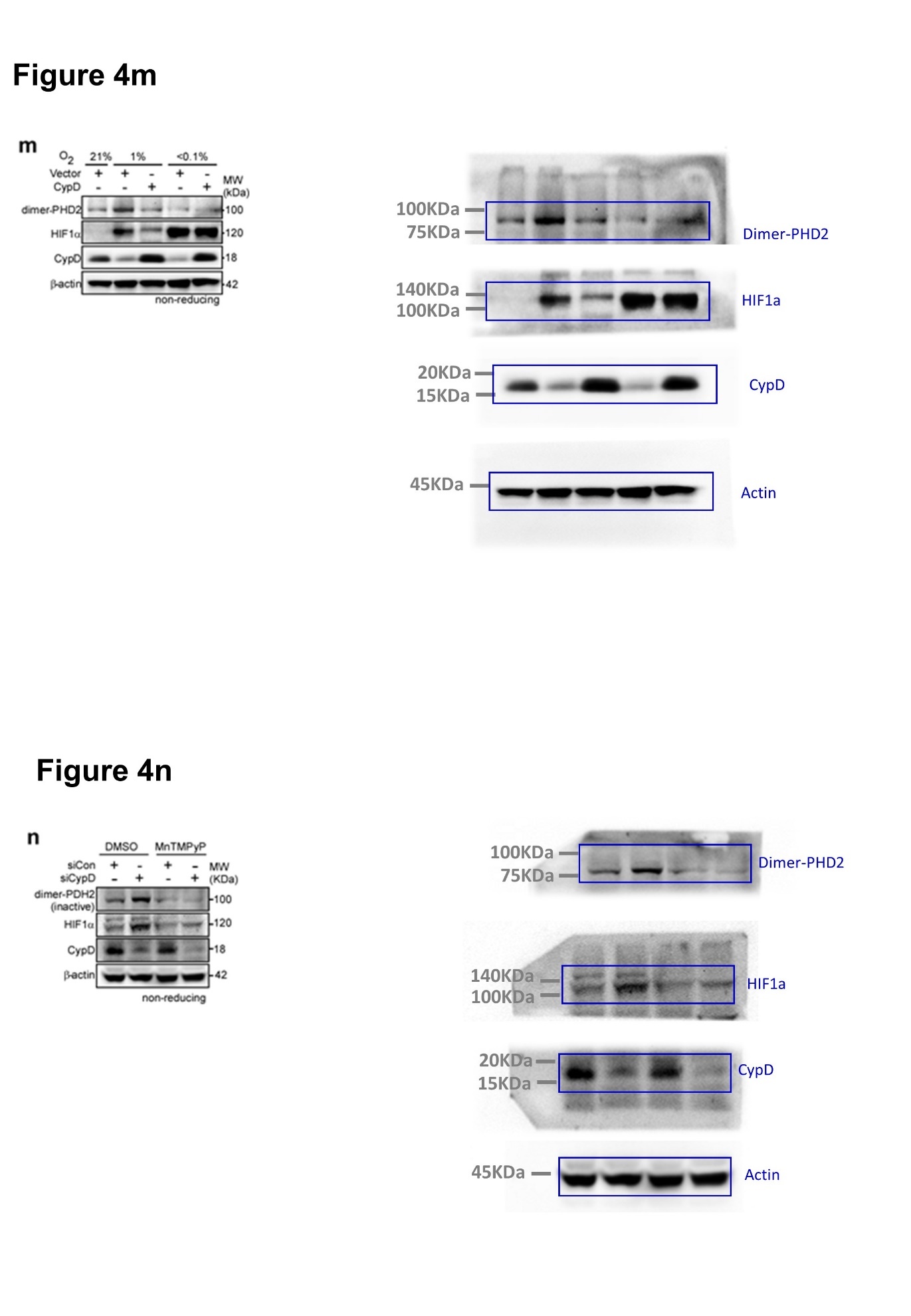


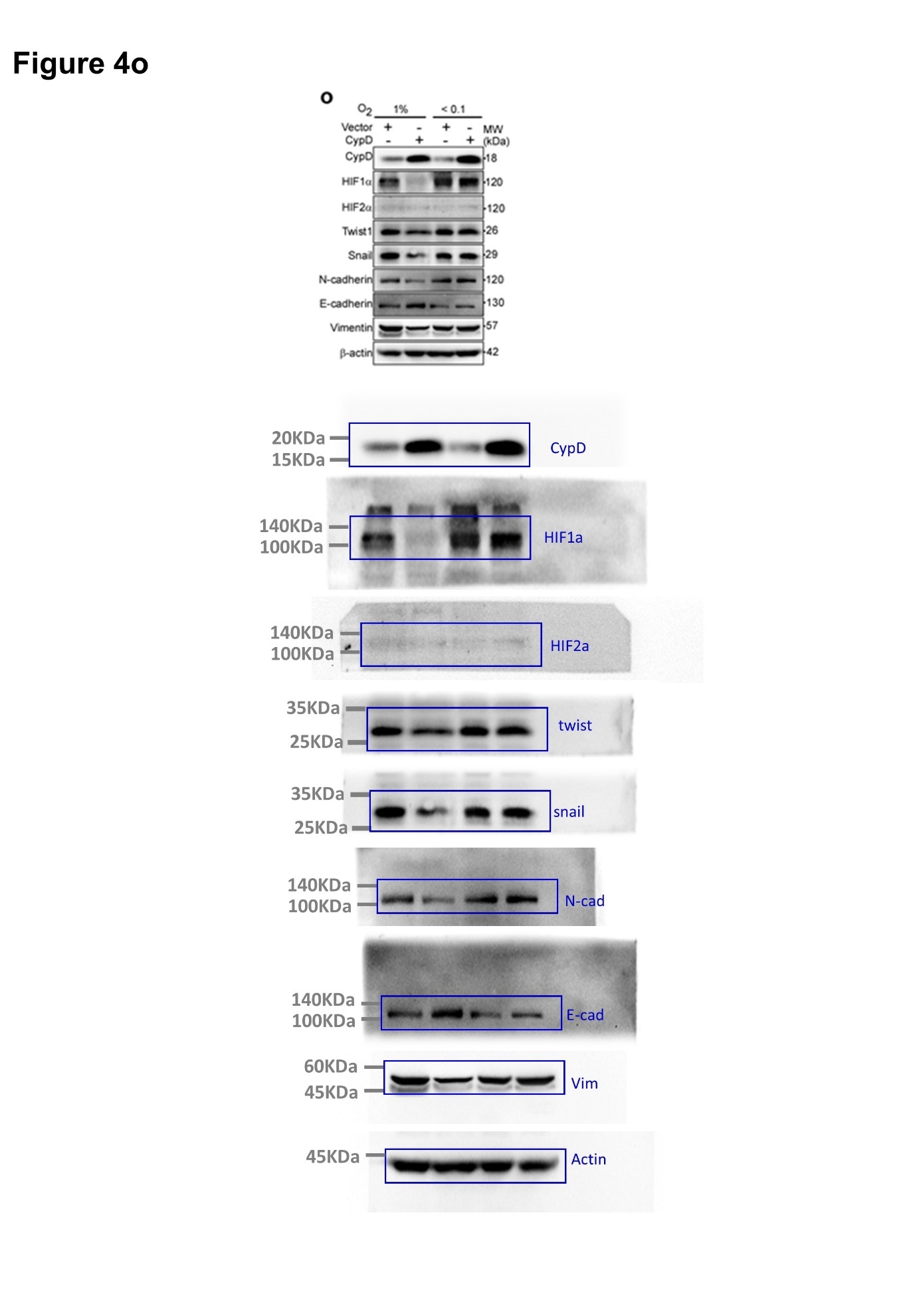


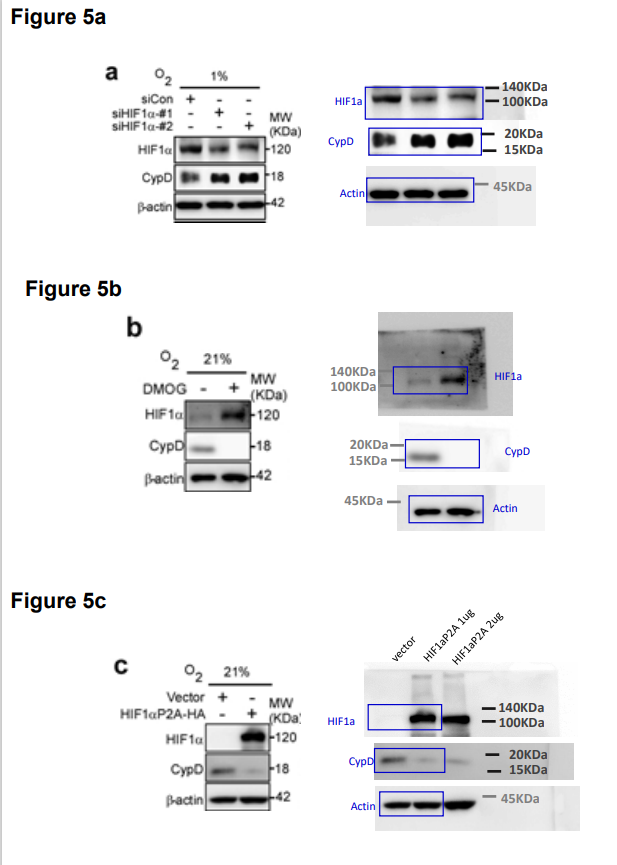


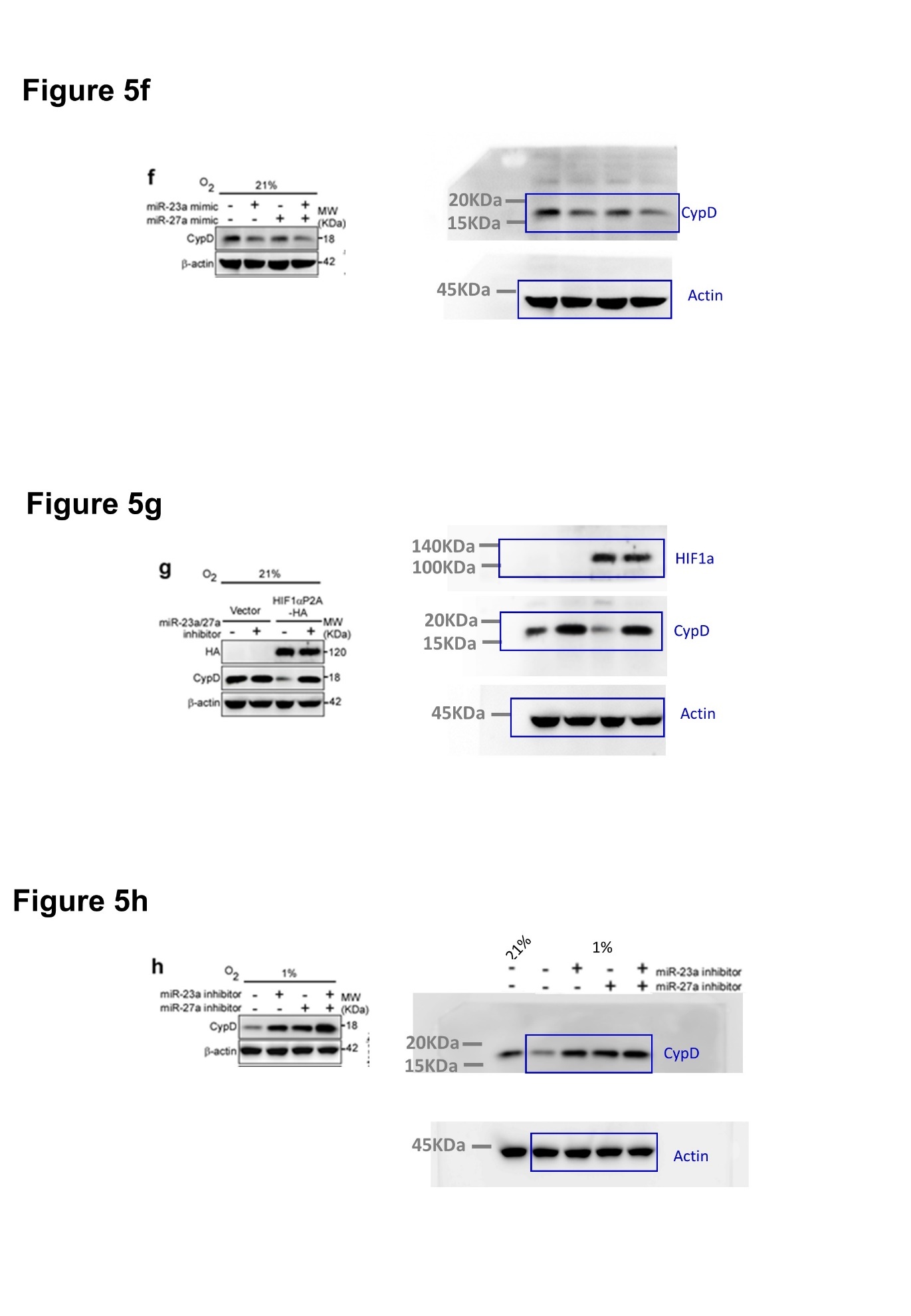


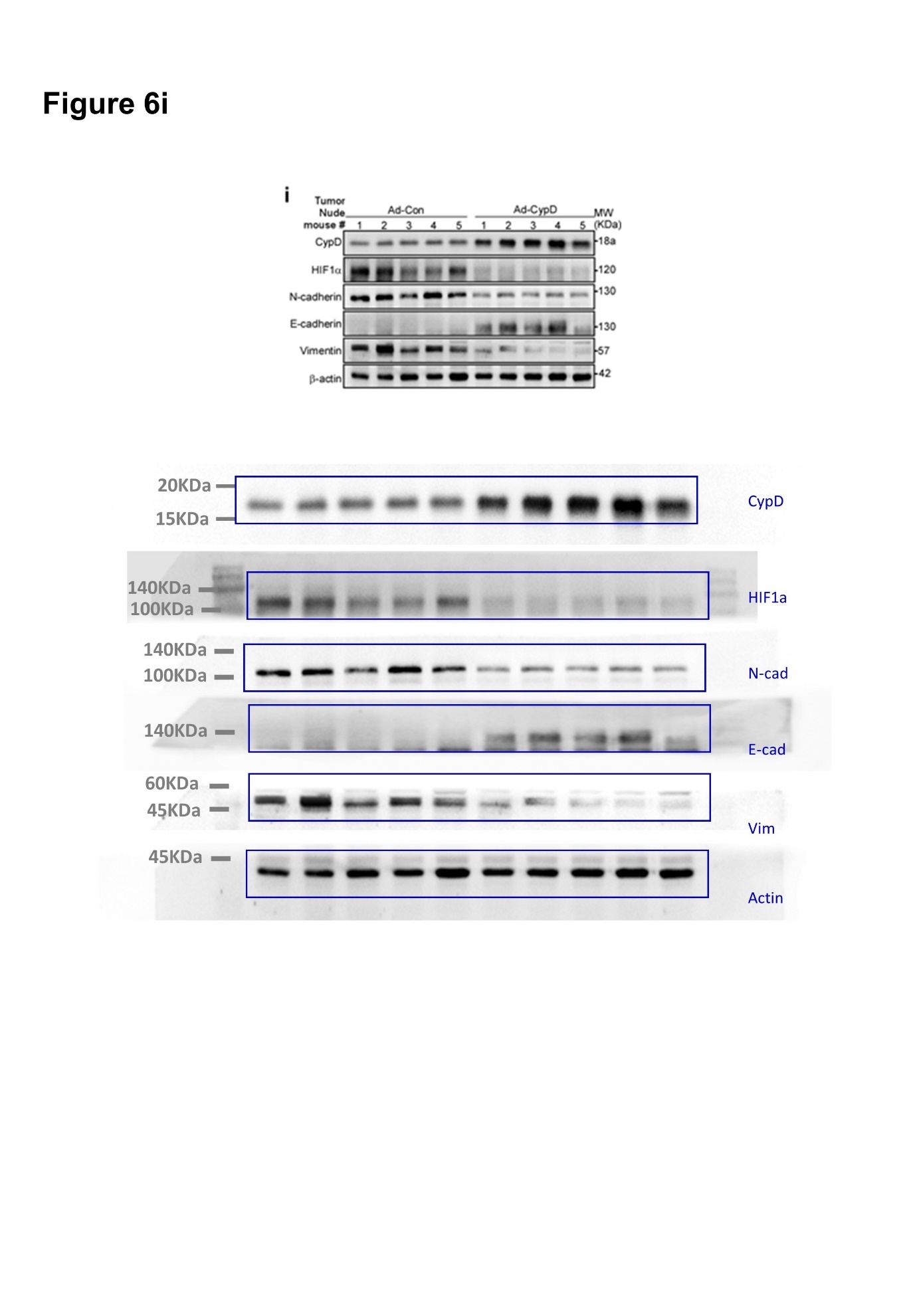


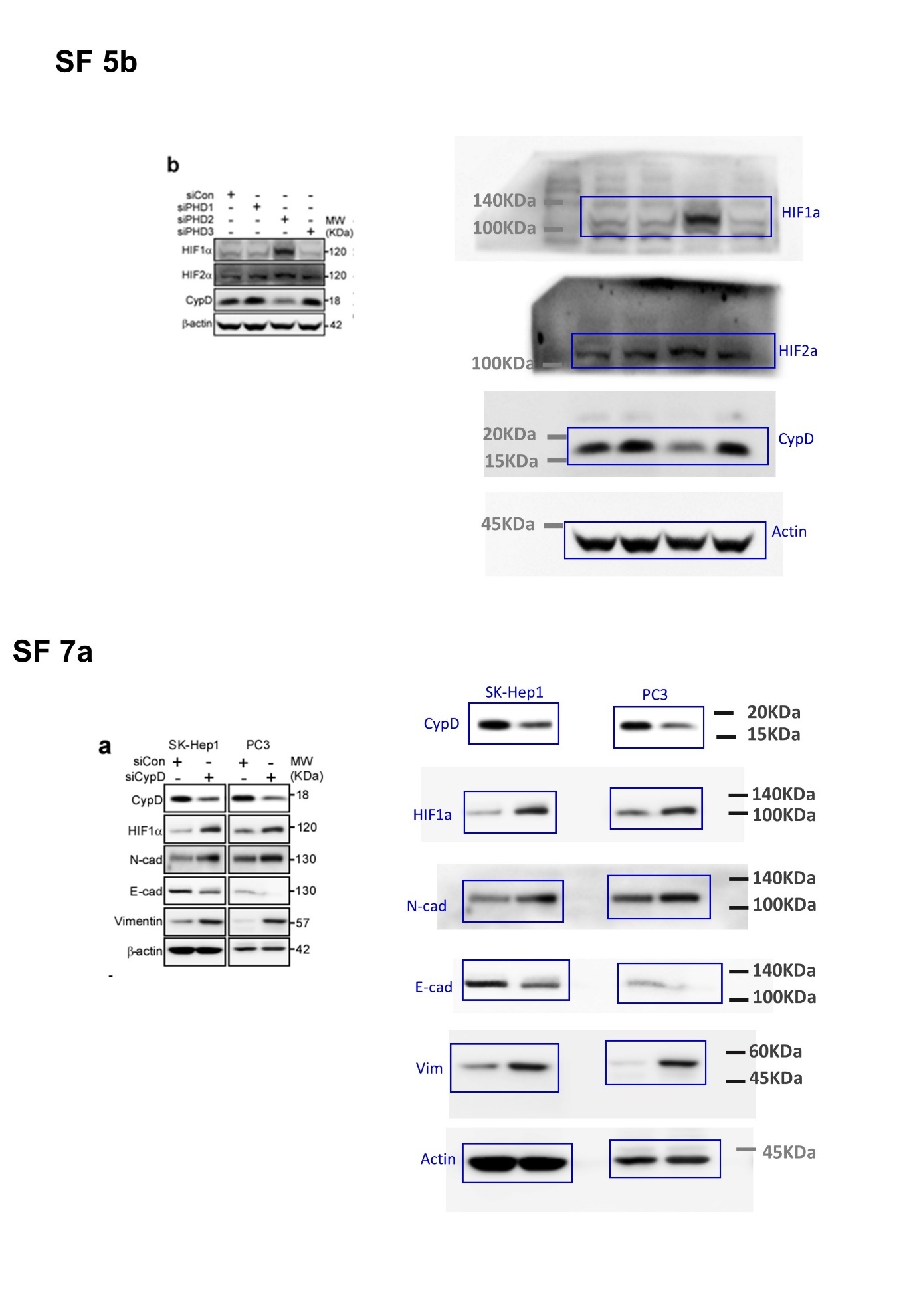


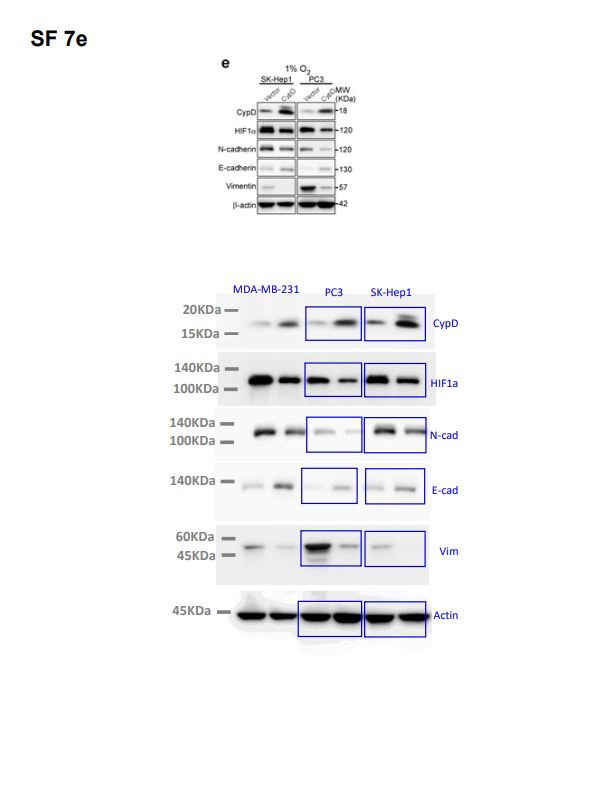


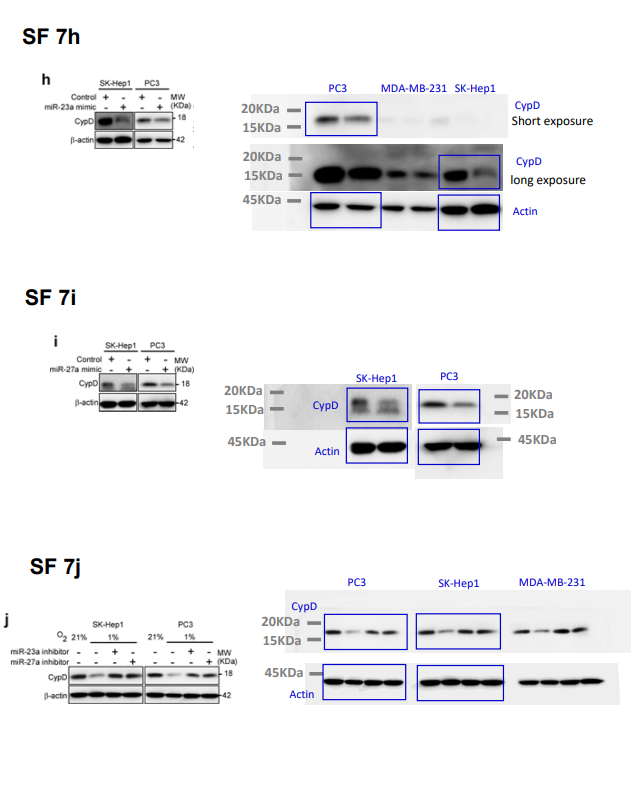


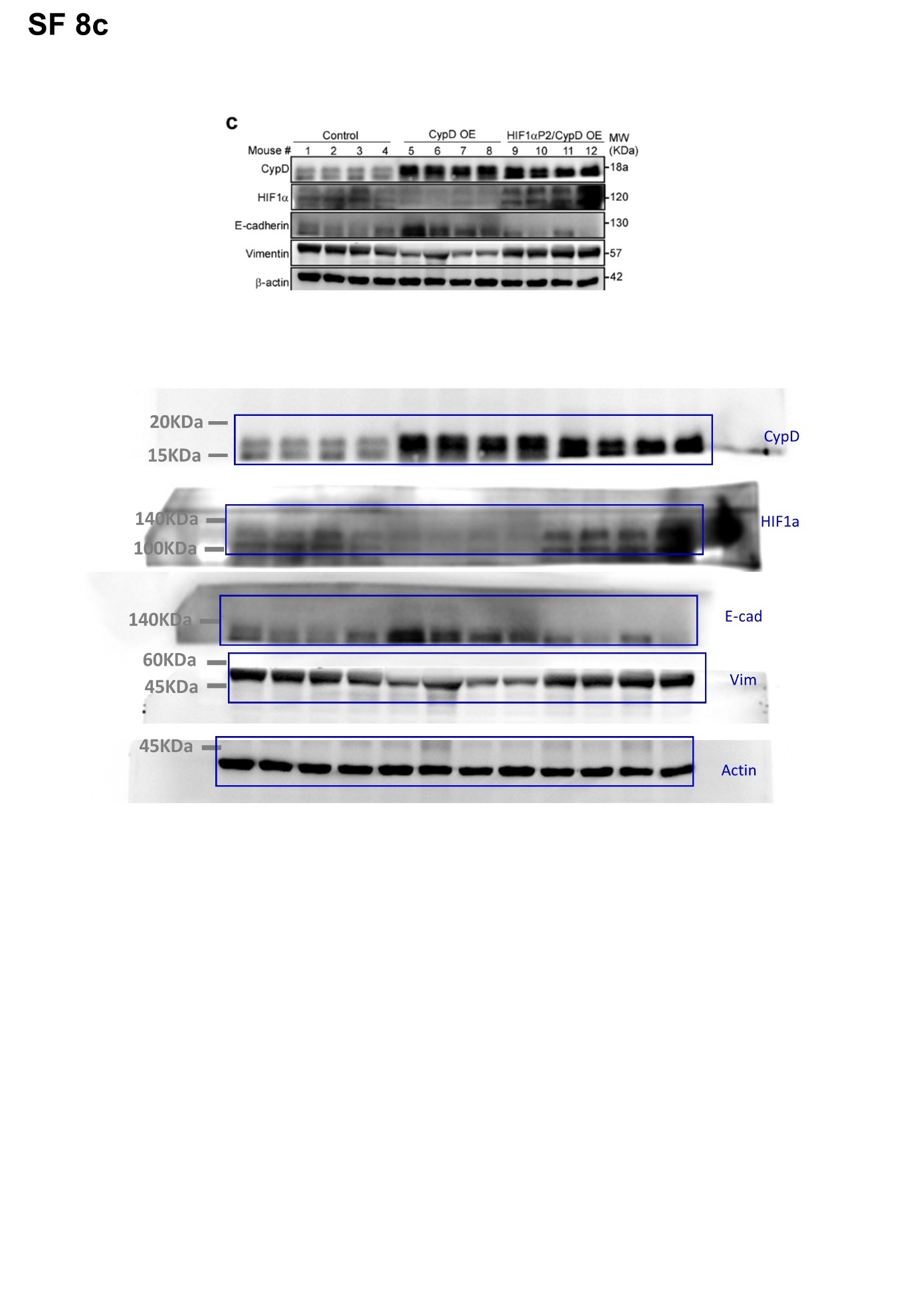


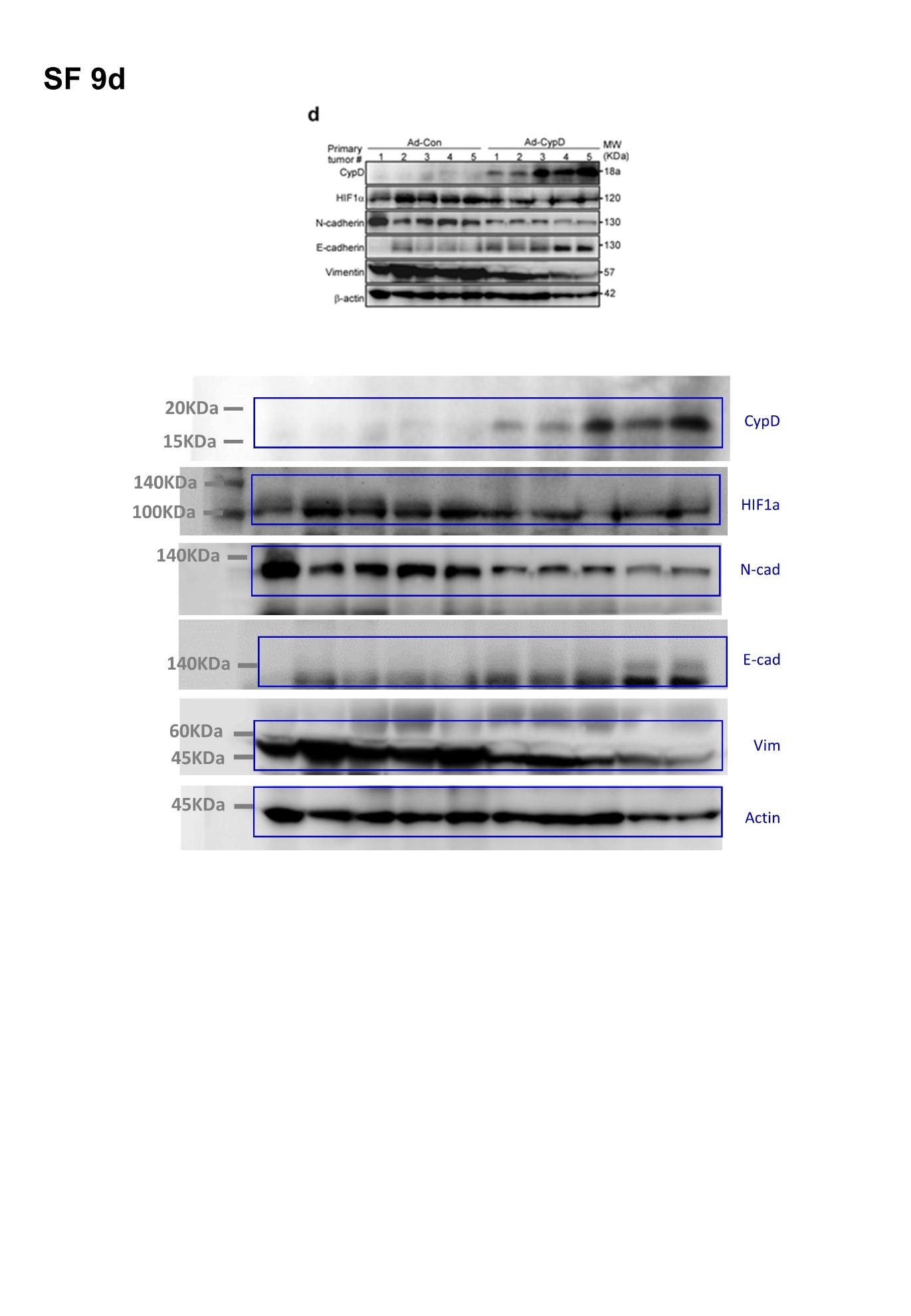


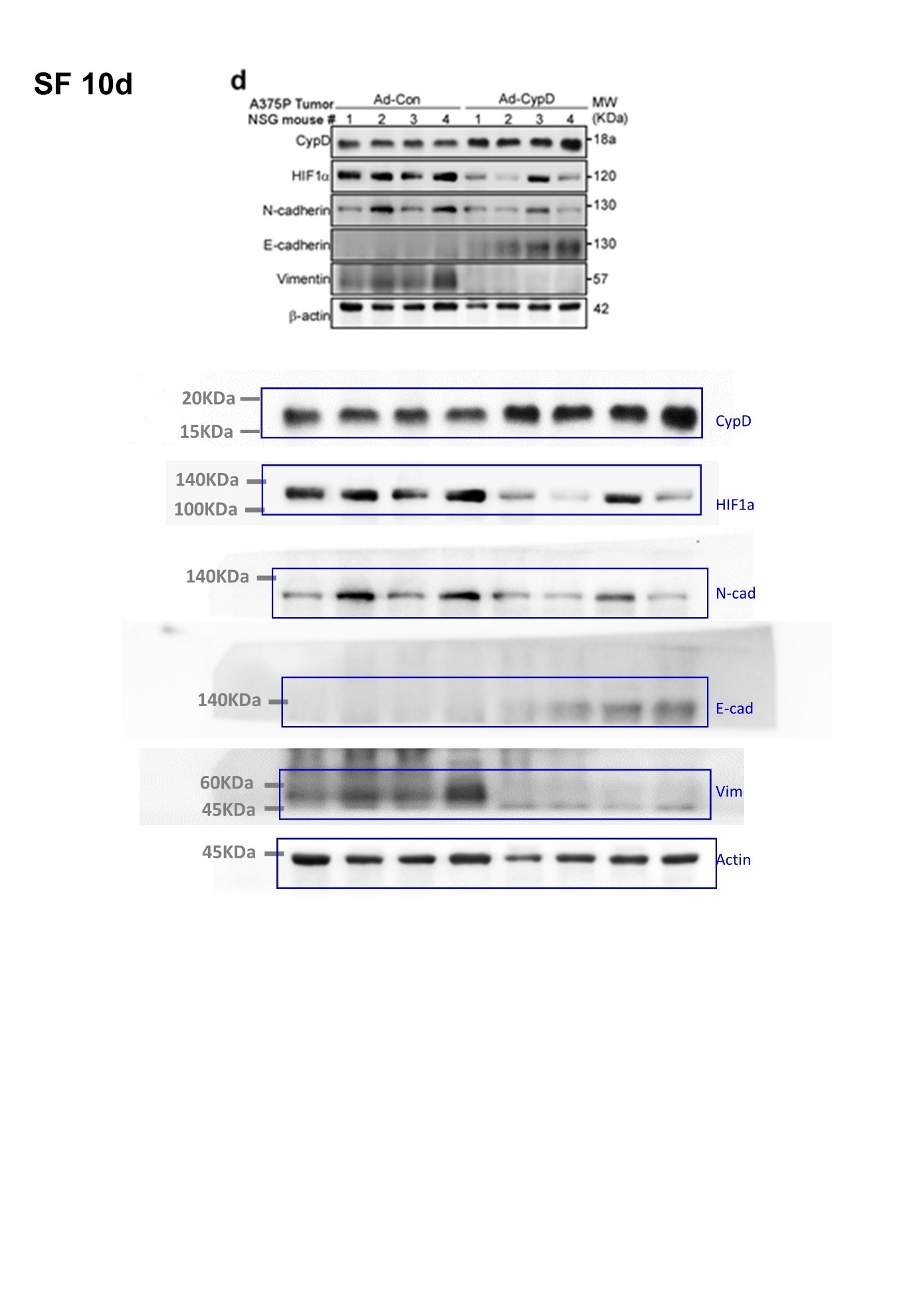


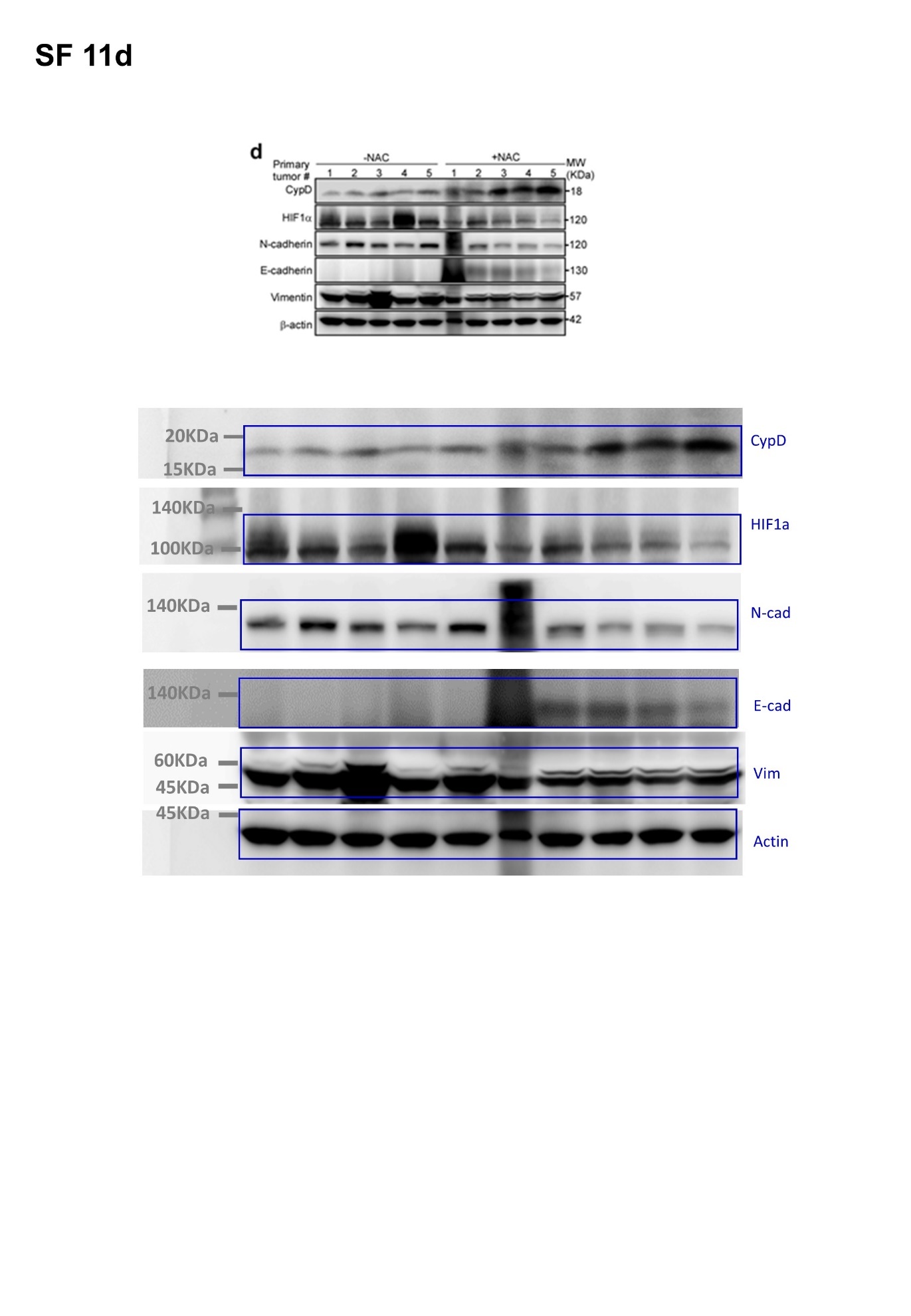

Supplement: Supplementary file 2 — Original immunoblot films [file 41392_2025_2314_MOESM2_ESM.docx]
